# Prediction of HIV-associated neurocognitive disorder (HAND) from three genetic features of envelope gp120 glycoprotein

**DOI:** 10.1186/s12977-018-0401-x

**Published:** 2018-01-27

**Authors:** Masato Ogishi, Hiroshi Yotsuyanagi

**Affiliations:** 1 Division of Infectious Diseases and Applied Immunology, Research Hospital, The Institute of Medical Science, The University of Tokyo, Tokyo, Japan; 2 National Center for Global Health and Medicine, Tokyo, Japan

**Keywords:** HIV-associated neurocognitive disorder (HAND), HIV envelope gp120 glycoprotein, Machine learning, Biomarker

## Abstract

**Background:**

HIV-associated neurocognitive disorder (HAND) remains an important and yet potentially underdiagnosed manifestation despite the fact that the modern combination antiretroviral therapy (cART) has achieved effective viral suppression and greatly reduced the incidence of life-threatening events. Although HIV neurotoxicity is thought to play a central role, the potential of viral genetic signature as diagnostic and/or prognostic biomarker has yet to be fully explored.

**Results:**

Using a manually curated sequence metadataset (80 specimens, 2349 sequences), we demonstrated that only three genetic features are sufficient to predict HAND status regardless of sampling tissues; the accuracy reached 100 and 94% in the hold-out testing subdataset and the entire dataset, respectively. The three genetic features stratified HAND into four distinct clusters. Extrapolating the classification to the 1619 specimens registered in the Los Alamos HIV Sequence Database, the global HAND prevalence was estimated to be 46%, with significant regional variations (30–71%). The R package *HANDPrediction* was implemented to ensure public availability of key codes.

**Conclusions:**

Our analysis revealed three amino acid positions in gp120 glycoprotein, providing the basis of the development of novel cART regimens specifically optimized for HAND-associated quasispecies. Moreover, the classifier can readily be translated into a diagnostic biomarker, warranting prospective validation.

**Electronic supplementary material:**

The online version of this article (10.1186/s12977-018-0401-x) contains supplementary material, which is available to authorized users.

## Background

Neurocognitive impairments during the course of chronic HIV infection, called HIV-associated neurocognitive disorder (HAND), remain as an unconquered clinical entity despite the improvement of combination antiretroviral therapy (cART) over the last 20 years [1, 2]. HAND is a comprehensive concept encompassing the broad spectrum of motor, cognitive, and neuropsychiatric impairment, in which persistent HIV infection in the central nervous system (CNS) plays a fundamental role. According to the criteria proposed by the HIV Neurobehavioral Research Center (HNRC), HAND is stratified into three conditions, namely, asymptomatic neurocognitive impairment (ANI), mild neurocognitive disorder (MND), and HIV-associated dementia (HAD) [3]. In a large cohort study from the U.S., prevalence estimates of ANI, MND and HAD were inferred at 33, 12 and 2%, respectively [4]. Other cohort studies yielded similar estimates [5–7]. Despite its wide prevalence, diagnostic and therapeutic strategies are quite limited; currently, there is no molecularly defined biomarkers, and prompt initiation of cART is the only clinically available treatment, though its effectiveness on preventing the progression of neurocognitive impairment is still in hot controversy [8, 9]. Indeed, as cART has become more accessible in resource-limited settings worldwide, thereby extending the expected lifespan of HIV-infected patients, the global burden of HAND is expected to be steadily on the rise. Recently, accumulating evidence suggests that persistent viral replication and ongoing diversification in the CNS compartment even in patients with undetectably suppressed viremia could lead to the emergence of neurotoxic quasispecies and thereby contribute to the progression of HAND [10, 11]. In this context, defining etiologically relevant diagnostic and/or prognostic biomarkers and optimal regimens based on those biomarkers in the era of modern cART is an inevitable step forward to improve current clinical practice.

Neurotoxic HIV viral quasispecies have been hypothesized to play an indispensable role in HAND pathogenesis. Although the mechanisms of HIV neurotoxicity has yet to be thoroughly clarified, several studies have suggested that there is a link between HAND and the neurotoxicity exerted by the orchestrated actions of several HIV proteins including trans-activating protein (*Tat*) and envelope glycoprotein (*Env*) [12]. Particularly, gp120, a fragment proteolytically cleaved from the *Env* protein, may mediate neuronal damage via direct induction of apoptosis both in rodents and primary human brain tissue culture [13–15]. On the other hand, hypervariable region 3 (V3) located at the middle of gp120 is primarily responsible for the genotypic and phenotypic diversity of HIV. A loop structure formed by V3 (V3 loop) interacts with chemokine coreceptors CCR5 and/or CXCR4, thereby determining multifaceted viral phenotypes including cell tropism [16]. Studies of CNS-derived viral isolates have indicated the links between CCR5 tropism, macrophage/microglia tropism, and the compartmentalization and persistent replication of viruses in the CNS [17–19]. Considering these insights, it is plausible to hypothesize that the gp120 glycoprotein serves as a primary, if not exclusive, determinant of both neurotropism, i.e., the ability to cross the blood–brain barrier and maintain replicative capacity in the CNS compartment, and neurotoxicity, i.e., the capability of igniting and/or fueling neurocognitive impairments. In this context, Pillai et al. [20] studied the C2V3 *env* subregion, and reported that the fifth residue of the V3 loop significantly correlated with neurocognitive deficit, although they did not explore the predictive significance of this signature. Indeed, a single amino acid signature is unlikely to be adequate to explain HIV adaptation during the course of HAND progression; thus, the combination of various signatures should be explored.

Machine learning (ML) is a highly promising technique for exploring a vastly large set of parameters to yield a potent classifier without prespecifying mathematical models. To gain optimized predictive accuracy, ML algorithms iteratively evaluate three types of error: training errors, validation errors (i.e., in-sample errors), and generalization errors (i.e., out-of-sample errors). The ultimate goal of ML-based prediction is to construct a classifier which has minimal generalization errors to unobserved real-world data. When a training dataset is provided, ML algorithms internally evaluate training errors to find the best set of parameters specific to the algorithm, and validation errors are evaluated by methods such as cross-validation (CV). When multiple algorithms with different sets of parameters are compared, the classifier with the smallest validation errors is selected. Then, the generalization errors should be evaluated with a testing dataset independent from model construction and selection.

Holman and Gabuzda applied ML-based approach to a manually collected metadataset of *env* C2V3C3 sequences derived from patients with or without HIV-associated dementia (HAD), reporting 75% accuracy for predicting HAD-associated *env* sequences [21]. Although their work provided intriguing insights into HIV neuropathogenesis, its generalizability is limited by several caveats. First, they reported the predictive accuracy via leave-one-out cross-validation. However, this corresponds to the validation error and may be a too optimistic estimation of the generalization error because of overfitting of the model against the training dataset. Rather, hold-out validation with no classifier retraining is necessary to correctly evaluate the generalization error. Second, although they only tested a simple rule-based classification algorithm, this could be outperformed by several recently implemented machine learning algorithms and an ensemble of those classifiers. Lastly, they attempted to construct a sequence-level classifier, and they empirically set a threshold at 95% of the patient’s sequences for classifying the patient as having HAD. However, such empirical criteria should be carefully interpreted for potential overfitting. Moreover, since it is plausible to assume that even patients with HAD harbor non-neurotoxic quasispecies, and vice versa for patients without clinically apparent neurocognitive impairments, a patient-level set of features capturing the diversity of intrapatient quasispecies could be more predictive rather than a sequence-level set of features.

The purpose of this retrospective analysis is to propose a potential biomarker for HAND. To this end, the most predictive genetic signatures were explored by generating an ML-based HAND prediction model. A thorough literature search led to the construction of the most comprehensive metadataset to date, comprised of 2494 *env* C2V3C3 sequences from 9 studies involving 85 specimens from 43 patients. Iterative ML and stepwise feature reduction yielded three genetic features. A final ensemble classifier achieved accuracy of 100 and 94% in the hold-out testing subdataset and as a whole, respectively. Specimens from various sampling sources were classifiable using the same genetic features. Clustering analysis stratified HAND into four distinct clusters. The datasets, the main analysis workflow, and the in-house functions were made publicly available so as to maximize the reproducibility of the entire work.

## Results

### Construction of annotated HIV *env* sequence metadataset

A large, curated sequence dataset annotated with relevant clinical information is indispensable for ML-based prediction of the HAND status. Initially, we considered using The HIVBrainSeqDB [22] (http://hivbrainseqdb.dfci.harvard.edu/HIVSeqDB/) or The HAND Database [23] (http://www.handdatabase.org/). However, because these databases did not seem comprehensive, we decided to conduct a manual literature review. A thorough literature search resulted in the construction of a manually curated metadataset derived from 9 studies involving 40 patients, and consisting of 2494 HIV *env* C2V3C3 sequences (see “Methods” section for details), among which 2358 were unique (Additional file 1: Table 1). Sequences isolated from HAND and NonHAND cases formed several phylogenetically distinct clusters (Additional file 1: Fig. 1). Supported by this observation, we decided to further explore ML-based approach to construct a classifier predicting the HAND status from the C2V3C3 sequences.

### Machine learning for predicting HAND status

The 2349 C2V3C3 sequences derived from HAND or NonHAND patients were converted into a numerical matrix using the 76 AAIndex schemes relevant to the physicochemical properties of amino acids. Patients diagnosed as either HIV-associated encephalitis (HIVE) or non-specific neuropsychiatric disorder (NPD) were excluded. Next, sequences were grouped by patient and sampling source, and representative statistics (e.g. mean and standard deviation) were calculated for each alignment position. Features with little variance, and a set of highly correlated features were excluded. In this manner, a total of 3169 patient-level predictive features were generated for 80 specimens. We performed ML with five distinct algorithms with ten different random seeds for hold-out data splitting. Stacking of the five classifiers was also attempted.

**Fig. 1:**
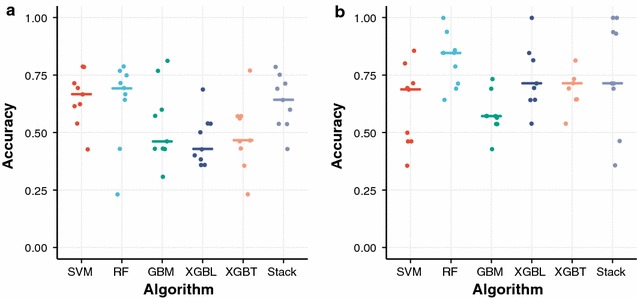
Performance of ML-based classifiers predicting HAND. The in-house metadataset constructed from an extensive literature search was divided into the training and testing subdatasets. Five ML classifiers and their stacked classifier were trained using the training subdataset, and their accuracies were evaluated using the testing subdataset. Stacking algorithm was XGBT. **a** Classifiers trained using all features. **b** Classifiers trained with three minimal features. Dots represent ML attempts with different random seeds. SVM, support vector machine; RF, random forest; GBM, gradient boosting machine; XGBL, extreme gradient boosting with linear booster; XGBT, extreme gradient boosting with tree booster; Stack, the stacked classifier

The mean and the best accuracy of the stacked classifiers trained using all features were 63 and 78%, respectively, in the hold-out validation subdataset (Fig. 1a and Additional file 1: Table 2). However, since this is extremely over-parametrized analysis, we attempted to reduce the number of features. First, algorithm-specific feature importance was calculated for each classifier, and the 20 most important ones were screened (Additional file 1: Table 3). Next, features selected by two or more distinct algorithms were selected. Finally, distributions between HAND and NonHAND cases were compared. *P* values were calculated by Welch’s *t* test, and adjustment for multiple comparisons was done according to the method controlling the false discovery rate (FDR) originally reported by Benjamini and Hochberg [24]. Features with adjusted *P* values of lower than 0.05 were retained. In this manner, seven important features were uncovered (Additional file 1: Fig. 2). Encouraged by the observation that feature reduction did improve the predictive accuracy, we further deciphered the best set of features by means of stepwise feature reduction (Table 1 and Additional file 1: Table 4). Surprisingly, we observed the highest accuracy being 100% in the testing subdataset in two different algorithms (Fig. 1b). These classifiers used only three features. The mean and the best accuracy of the stacked classifiers were 76 and 100%, respectively (Fig. 1b and Additional file 1: Table 5). The accuracy of the best stacked classifier for the whole dataset was 94%, where the HAND status was predicted correctly in 75 out of 80 specimens. The distributions of Bayesian posterior probabilities showed that this classifier is expected to work well when the prior probability of HAND lies within the range from ~ 25% to ~ 50% (Additional file 1: Figure 3). Finally, as an external validation, all of the three specimens obtained from two patients with NPD were classified as HAND. One specimen was obtained from Patient 196, who was not diagnosed as HAD due to lack of information in the original paper despite the evidence of neuropsychiatric impairment [25]. The other two specimens were obtained from Subject 7115 at July 8th, 2002 [26]. Although the diagnosis of HAD could not be made due to confounding conditions at that moment, the same patient was diagnosed as moderate to severe HAD 2 years later. These two cases highlight the potential utility of our sequence-based HAND classifier as a diagnostic aid.

**Table 1:** Important AAIndices identified in this study

AAIndex ID	Description	Reference
DIGM050101	Hydrostatic pressure asymmetry index	Di Giulio [47]
KARP850102	Flexibility parameter for one rigid neighbor	Karplus and Schulz [48]
BHAR880101	Average flexibility indices	Bhaskaran and Ponnuswamy [49]

For further information regarding each AAIndex, visit the AAIndex website (http://www.genome.jp/aaindex/) [42]

**Fig. 2:**
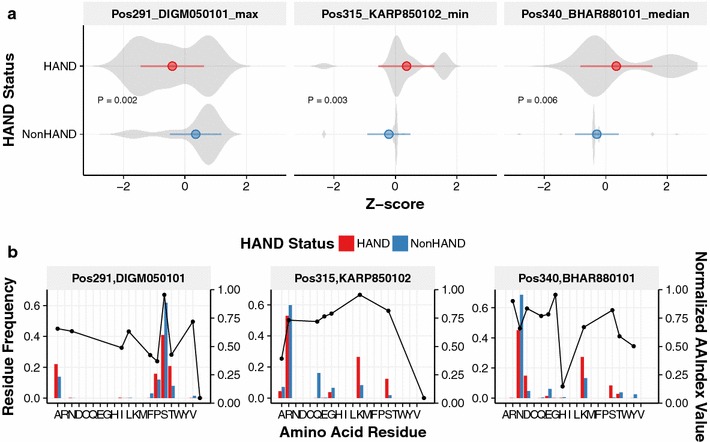
The minimal set of features predictive of HAND. Model-specific feature importance was estimated using the *varImp* function implemented in the *caret* package for each of the ML algorithms except SVM. Features listed in the top 20 in two or more algorithms were selected. *P* values were calculated using Welch’s *t* test and adjusted by the FDR-based method [24]. Adjusted *P* values of less than 0.05 were considered significant. In this manner, seven genetic features were retained (Additional file 1: Figure 2). Stepwise feature reduction was performed, and the minimal set of features yielding the best-performing stacked classifier was obtained. **a** Distributions of detected features among HAND and NonHAND groups. The values of each feature were converted to Z-score for visualization purposes. **b** Scaled AAIndex values and relative residue frequencies in sequence sets derived from HAND and NonHAND cases. The weights of individual sequences are normalized by the respective sequencing depths of individual patients. The alignment position numbers correspond to the positions in the HXB2 HIV-1 sequence (accession: K03455)

The distributions of the most important features were significantly different between the HAND and NonHAND cases (Fig. 2a). At position 291 (Pos291), the maximum value of the AAIndex DIGM050101, which represents a hydrostatic pressure asymmetry index, was shown to be the most predictive. The difference between the HAND and NonHAND cases was explained by the decrease of the frequency of 291S in the HAND group (Fig. 2b). Meanwhile, the other two important AAIndices, namely, KARP850102 and BHAR880101, are related to the flexibility of the residue. Variants enriched in the HAND cases such as 315 K/S and 340D/K/S contributed to the different feature distributions (Fig. 2).

### Molecular stratification of HAND through the minimal set of genetic features

**Fig. 3:**
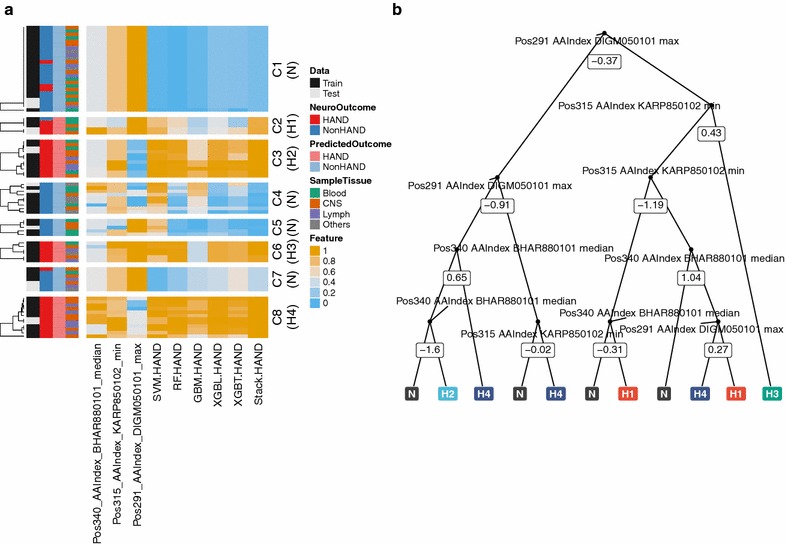
Molecular stratification of HAND. **a** K-means clustering analysis. The three genetic features and the predicted probabilities from the six classifiers were used for k-means clustering analysis. Eight clusters were identified by inspecting the dendrogram manually. HAND-rich clusters, namely, C2, C3, C6, and C8, were renamed as H1, H2, H3, and H4, respectively. Other clusters were labeled as N. Heatmap was constructed using the *ComplexHeatmap* package [45]. **b** The representative decision tree for predicting the HAND clusters. The three genetic features were used to construct a multiclass RF classifier for the four HAND clusters and N. The number of nodes in the decision trees ranged from 13 to 29. The representative tree with the median number of nodes, 23, was shown. SVM, support vector machine; RF, random forest; GBM, gradient boosting machine; XGBL, extreme gradient boosting with linear booster; XGBT, extreme gradient boosting with tree booster; Stack, the stacked classifier

HAND is a diagnosis of exclusion, thus inherently harboring some heterogeneity. We noticed that, although the stacked classifier predicts the HAND status with high confidence, the subordinate classifiers returned considerably varied probabilities to the same cases, indicating that each of the classifiers captures distinct aspects of the triad of the genetic features. Indeed, clustering analysis revealed four distinct HAND-rich clusters (H1-H4) with characteristic genetic landscapes (Fig. 3a). Clusters enriched with NonHAND cases were combined as N. Random forest algorithm successfully constructed a classifier for these five categories, with the estimated accuracy from internal CV being 94%. A representative decision tree with the median number of nodes was shown in Fig. 3b. The tree understandably reflects the similarities between H2 and H4, and those between H1 and H3. When each amino acid variants were considered to be features, no apparent cluster-specific enrichment occurred, underscoring the effectiveness of our AAIndex-based feature generation framework (Additional file 1: Fig. 3).

**Fig. 4:**
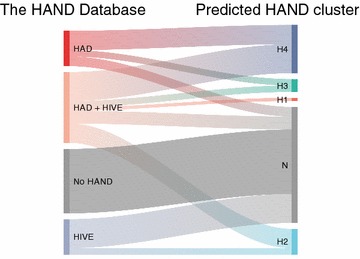
HAND clusters among the cases registered in The HAND Database. A total of 1687 *env* sequences from 68 specimens were retrieved from The HAND Database [23]. The RF classifier (Fig. 3b) was applied to predict the corresponding HAND clusters. HAD, HIV-associated dementia; HIVE, HIV encephalitis

To unveil the characteristics of each of the HAND clusters, we next applied the random forest classifier to the entire dataset obtained from The HAND Database (http://www.handdatabase.org/), which is a recently published, manually curated database of HIV sequences with clinical metadata [16] (Fig. 4). Collectively, 26 out of 33 (79%) HAD cases were classified into one of the HAND clusters, whereas all of the twenty cases originally annotated as not neurocognitively impaired were predicted as such (100%). Interestingly, nine out of eleven (82%) HIVE cases were predicted as N. Moreover, remaining two HIVE cases and the 6 cases with HAD and overlapping HIVE were classified as H2. These observations highlight the uniqueness of H2, potentially bridging the two distinct disease entities, namely, HAND and HIVE.

### Estimation of the global burden of HAND

One major obstacle against the epidemiological study regarding HAND is the dearth of molecularly defined biomarkers. Currently, a careful neuropsychiatric examination is the only solid basis; in addition to this, various tests including brain CT/MRI and the cerebrospinal fluid (CSF) analysis are frequently required to exclude various mimicking diseases such as meningoencephalitis, toxoplasmosis, and primary CNS lymphoma. Biomarkers measurable from peripheral plasma could greatly reduce the burden for diagnostic procedures, and thereby facilitate epidemiological and other clinical studies particularly in resource-limited settings.

**Fig. 5:**
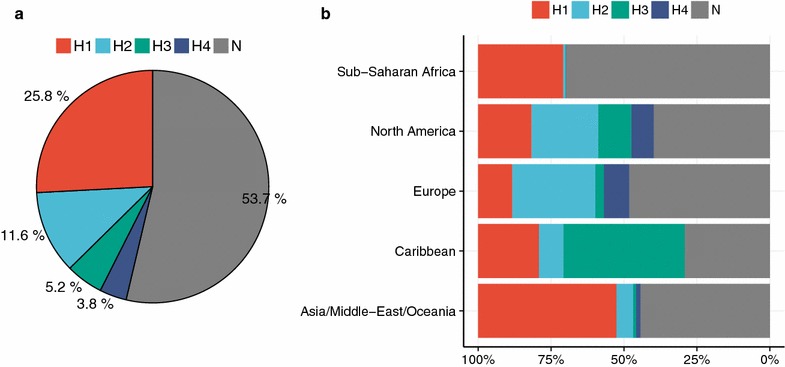
Estimation of the global burden of HAND. A total of 19,800 *env* sequences from 1619 specimens were retrieved from The Los Alamos HIV Sequence Database. The RF classifier (Fig. 3b) was applied to predict the corresponding HAND clusters

In view of this application, we retrospectively estimated the global burden of HAND from the entire sequence dataset deposited in the Los Alamos HIV Sequence Database (https://www.hiv.lanl.gov/content/sequence/HIV/mainpage.html), the largest database to date. Collectively, 46% of the cases were predicted as HAND, which was slightly higher than estimates from historical cohorts [4–7] (Fig. 5a). Among the predicted HAND cases, H1 was the most common cluster, followed by H2, H3, and H4 (Fig. 5a). Geographically dissecting, Caribbean region was the region with the largest predicted HAND burden (71%), whereas Sub-Saharan Africa was the lowest prevalence (30%). Among HAND clusters, H1 was dominant in Sub-Saharan Africa and Asia/Middle-East/Oceania (97 and 85%, respectively). In other regions, no single dominant cluster was noted. Caribbean region was characterized by the high prevalence of H3 (59%). Europe and North America regions had relatively high prevalence of H2 (54 and 38%, respectively). These regional differences in viral genetic landscape might be associated with various factors such as ethnicity and human leucocyte antigen (HLA) allele frequencies. Among the HAND clusters, H1 had a moderately higher viral load and lower CD4^+^ T-cell count compared to H2. Meanwhile, H3 had a statistically higher CD4^+^ T-cell count compared to H1 (Additional file 1: Figure 5). These trends were unchanged even when the *P* values were adjusted according to the FDR-based method [24].

### Data and code availability for future research

Both the datasets and the in-house functions created in this study were bundled as the R package *HANDPrediction*, and distributed on GitHub (https://github.com/masato-ogishi/HANDPrediction). To facilitate future research, the entire analysis workflow was also publicly distributed as an HTML document (Additional file 2).

## Discussion

In this work, the three genetic features of the HIV *env* gene most predictive of the HAND status were identified through the construction of a highly accurate classifier via machine learning (ML). The surprisingly small number of features, three, strongly counter-argues the possibility of overfitting and supports the generalizability of the model to external datasets. The set of features stratified the 37 specimens derived from HAND cases into four clusters. The stratification process was successfully recapitulated by random forest algorithm, which enabled extrapolation of the genetic feature-based classification of HAND status. Estimation of global burden of HAND was demonstrated using the Loa Alamos HIV sequence database. The regional differences in the relative frequencies of HAND clusters probed by this retrospective analysis underscore the potential usefulness of our framework as an aid for epidemiological research, thereby warranting prospective validation.

In contrast to previous studies, neurotoxicity was stringently distinguished from neurotropism during the construction of the metadataset in this study. This is because it is inappropriate to discuss those two distinct phenotypes interchangeably, since neurotoxic viral quasispecies that may trigger neurocognitive impairment could reside both inside and outside the CNS, and viral quasispecies harbored in the CNS do not necessarily exert neurotoxicity. Indeed, as shown in Fig. 3a, HAND-associated genetic signatures were shared among specimens derived from the CNS, lymphatic system, and peripheral circulation. This indicates that selection pressure outside the CNS is not a major driver for quasispecies evolution, which is consistent with a recent observational study led by Stefic et al. [27].

It is an exciting possibility that viral sequences obtained from peripheral circulation could be used as a diagnostic biomarker of HAND. Whether these genetic biomarkers provide clues to HAND at asymptomatic stage is of great interest, as many neuropsychiatric tests suffer from lower diagnostic performance at this stage [28, 29]. However, one caveat of this study is that the sequences were mainly obtained from AIDS patients without viremia suppression by modern cART. In contrast, prompt initiation of cART is the gold standard of contemporary clinical practice [30]. In this setting, immune reconstitution due to cART may affect viral quasispecies with HAND-associated signatures and alter their systemic distributions. Meanwhile, CNS penetration effectiveness score of cART compound is another consideration, since higher penetration score has been associated with lower neurocognitive impairment [31]. However, how the architecture of HIV quasispecies is affected by various cART regimens, and what roles these alterations may play in the pathogenesis of HAND, should be elucidated in future research.

Patient-level features are more informative than sequence-level features for predicting patient-level phenotypes [32]. Consistent with this viewpoint, the summary statistics representing the distribution of physicochemical properties of intrapatient viral quasispecies were used as the features on the basis of which ML was performed. One caveat of this approach is the sequence depth per patient; observed relative frequencies of each of the amino acid variants at each of the positions may not reflect true intrapatient abundance with limited sequencing depth. Alternatively, next-generation sequencing platform could allow researchers to estimate relative abundance of variants with remarkably improved accuracy. We have previously shown that intrapatient abundances of viral quasispecies could be reliably estimated bioinformatically from short-read sequence datasets generated by the Illumina MiSeq platform [33]. This process is known as “quasispecies reconstruction”. Integration of high-throughput sequencing technology and quasispecies reconstruction could enable more accurate estimations of intrapatient quasispecies abundance with augmented scalability. Such large-scale datasets could bolster the precision and accuracy of the HAND prediction framework presented in this work.

A number of gp120 variants have been associated with neurotropism and/or neurotoxicity. For example, Dunfee et al. [34] reported T283N as a neurotoxic variant causing enhanced macrophage infectivity and neuronal degeneration. Duenas-Decamp et al. [27] showed that the otherwise non-macrophage-tropic strain LN40 can be transformed into a macrophage-tropic strain by introducing 283 N substitution. However, in an already macrophage-tropic strain (B33), substitution of 283 N into 283T did not alter tropism, indicating the existence of other determinants [27]. In our analysis, three positions, namely, Pos291, Pos315, and, Pos340, were identified to be the most predictive for HAND status (Fig. 2b). Holman and Gabuzda also reported the involvement of Pos315 in HAND-predicting signature [21]. Pos315 resides in the tip of the V3-loop, and various variants such as R315K, R315T, and R315Q have been associated with reduced efficacy of neutralizing antibodies (NAbs) [35–38]. In our analysis, R315K and R315Q were enriched in the HAND and NonHAND cases, respectively (Fig. 2b). Although the other two positions, Pos291 and Pos340, were less intensively studied, S291 (enriched in NonHAND) has been associated with decreased infectivity to macrophages in R5 virus [27]. Meanwhile, compartmentalization of N340, a variant enriched in NonHAND in our analysis, to the CNS was observed in some cases [39]. Both S291 and N340 were also identified in this study (Additional file 1: Figure 4).

The current concept of HAND is heterogeneous due to its nature of being diagnosed on the basis of symptomatic criteria and by exclusion of other confounding conditions. To our knowledge, there is no attempt to date to molecularly stratify the disease entity. In this work, four HAND clusters were identified based on a clustering analysis. Particularly, H2 is interesting because it was associated with HIVE (Fig. 4). Since H2 and the closest cluster H4 were distinguished by the Pos340 feature (Fig. 3), and H4 was associated with both HAD and HAD + HIVE (Fig. 4), Pos340 might be important in separating HAND and HIVE. Moreover, geographically speaking, both H2 and H4 seemed to be enriched in Europe and North America (Fig. 5). Such geographical difference, if is the case, should be taken into consideration when interpreting various research on HAND from various nations. The biological and epidemiological relevance of those variants and clusters has yet to be elucidated, thus warranting further research.

This study has some limitations, similarly to prior studies. First, since this is a retrospective observational study, no causative link can be definitively established. Amino acid signatures detected could be relevant to the neurotoxicity of HIV, but should not be interpreted as causative of HAND. Second, although unprecedented size, the numbers of unique specimens and patients were fairly small. Although we successfully reduced the number of required genetic features down to three, the risk of overfitting to the entire dataset should not be negated. Prospective collection of the adequate size of specimens would be the only strategy to effectively resolve this concern. Third, since most of the currently available *env* sequences were derived from HAD cases, the most severe form of HAND, the utility of our analysis in predicting early-stage HAND has yet to be fully verified. Similarly to this point, the effect of cART regimens on the evolutionary trajectory of viral quasispecies should also be taken into consideration in future research. We do not argue that our analysis provides all answers; rather, we hope this work could be a starting point. Therefore, we made publicly available the datasets, the custom codes, and the entire analysis workflow for the community.

## Conclusions

In this study, robust prediction of HAND status from three genetic features derived from the HIV *env* sequences was demonstrated. Furthermore, based on the combination of these three genetic features, we stratified HAND into four clusters with unique characteristics. These results could be utilized as a diagnostic aid after prospectively validation. Finally, the biological and epidemiological significance of newly discovered genetic features, potentially providing the basis of the development of novel cART regimens specifically optimized for HAND-associated quasispecies, are to be elucidated in future research.

## Methods

### Computational analysis

All computational analyses were conducted using R ver. 3.4.1 (https://www.r-project.org/) [40]. The latest versions of R packages were consistently used. The dataset and the scripts generated in this study are available as the R package *HANDPrediction* on GitHub (https://github.com/masato-ogishi/HANDPrediction). The entire analysis workflow is also available as an HTML document (Additional file 2).

### Assembly of the HIV *env* sequence metadataset

A thorough literature search was conducted to collect previously published studies on HIV neurotoxicity and/or neurotropism. Sequences and accompanying metadata were retrieved from the Los Alamos HIV Sequence Database (http://www.hiv.lanl.gov/content/sequence/HIV/mainpage.html/) and manually curated. Diagnoses of HIV-associated neurological conditions were retrieved from original publications for all of the cases. The sub-categories of HAND (AMI, MND, and HAD) were combined as ‘HAND’, and the AIDS-dementia complex (ADC) was also considered ‘HAND’ in this study. HIVE and other NPDs were labeled as such. Cases with no neurocognitive impairments were labeled as ‘NonHAND’ regardless of other CNS diseases including bacterial meningitis, toxoplasmosis, and CNS lymphoma. The sample sources were categorized into one of the following categories: ‘CNS’, ‘Blood’, ‘Lymph’, and ‘Others’.

### Alignment of HIV *env* sequences

The HXB2 HIV-1 sequence (accession: K03455) was used as a reference. The *env* region was identified by mapping sequences to the reference sequence using Geneious ver 8.1.8 (www.geneious.com). The built-in Geneious mapper was used with the “Medium Sensitivity” option selected. Default parameters were used. Sequences not mapped to the reference were discarded from the metadataset. The *env* C2V3C3 regions were manually determined, clipped, and re-aligned with MAFFT [41]. The alignment was refined and translated using the HIVAlign tool with the HMM-align option selected (https://www.hiv.lanl.gov/content/sequence/VIRALIGN/viralign.html). Alignment gaps shared by the reference sequence and more than 75% of the aligned sequences were manually removed. Sequences containing stop codons inside the C2V3C3 region were discarded.

### Machine learning

#### AAIndex matrix

AAIndex metrics (http://www.genome.jp/aaindex/) [42] were adopted as quantitative measures of biophysicochemical properties of each amino acid. A total of 531 AAIndices were retrieved from the *BioSeqClass* package available in *Bioconductor* [43]. The 76 AAIndices whose names matched with one of the following phrases were selected for machine learning: ‘Hydro’, ‘Charge’, ‘Polar’, ‘Distribution’, and ‘Flexi’. A C2V3C3 sequence was converted to a numerical vector comprising a set of AAIndex values corresponding to each amino acid residue at each alignment position. For all gaps and ambiguities (i.e., two or more amino acid residues indicated), values for all AAIndices were set to zero. In this manner, all sequences were converted to a numerical matrix, which had 76 × 189 (188 alignment positions plus one gap) columns.

#### Hold-out validation

The metadataset was split into the training and testing subdatasets at a ratio of 4:1. Note that the metadataset was split at the patient level, not at the sequence level. Sequence-level data splitting is inappropriate because the HAND vs NonHAND status is assigned to patients, not to individual sequences, and the genetic relatedness of the sequences derived from the same patient will likely lead to biased classification.

#### Preprocessing

In the training subdataset, columns with zero variance and near-zero variance were removed using the *preProcess* function with the ‘zv’ and ‘nzv’ method implemented in the *caret* package [44]. Then, highly correlating columns were filtered using *preProcess* with the ‘corr’ method. After these filtration steps, 3169 unique features were retained. Finally, the features were centered and scaled using *preProcess* with the ‘center’ and ‘scale’ methods. All preprocessing steps were carried out with default parameter settings. In the testing phase, the same preprocessing conditions prepared in the training phase were applied.

#### Machine learning with different algorithms

For simplicity, binary classification was attempted, i.e., HAND vs NonHAND. The following algorithms were compared for performance: support vector machine (SVM), random forest (RF), gradient boosting machine (GBM), extreme gradient boosting with linear booster (XGBL), and extreme gradient boosting with tree booster (XGBT), all of which are implemented in the *caret* package. “Stacking” of the classifiers was done using XGBT as a supervised learning algorithm. Ten-fold repeated three-fold CV was conducted in the training phase to improve the generalizability of the classifiers. Their predictive performances, i.e., sensitivity, specificity, and overall accuracy, were estimated using the testing subdataset.

#### Feature importance analysis

Model-specific feature importance was estimated using the *varImp* function implemented in the *caret* package. All models except SVM tested in this study have their own feature importance measures. The 20 most important features from each of the models were combined, and features detected in two or more different models were selected. Next, the distribution of the feature values among the HAND and NonHAND groups were compared by Welch’s *t* test, and *P* values were adjusted by the FDR-based method by Benjamini and Hochberg [24]. Features whose adjusted *P* values were less than 0.05 were selected. Finally, stepwise feature reduction was iteratively performed. ML was performed on the training subdataset with one of the features removed, and the accuracy in the testing subdataset. The removed feature giving the highest accuracy of the stacked classifier was removed for the next iteration.

### Clustering analysis

*K*-means clustering was performed on the minimal set of the most important features, and the predicted probabilities by each of the classifiers. Visualization of the heatmap and dendrograms were performed using the *ComplexHeatmap* package [45]. Clusters enriched with the HAND cases were identified by manual inspection of the dendrogram. Clusters enriched with the NonHAND cases were combined and labeled as ‘N’. The minimal set of the most important features was used to construct a multiclass random forest classifier classifying the HAND clusters and N using the entire dataset.

### Characterization of HAND clusters using The HAND Database

The HAND Database [23] (http://database.handdatabase.org/) was used to characterize each of the HAND clusters. The entire dataset was downloaded as is. A total of 1687 *env* sequences from 68 specimens were obtained. Sequences were aligned to the HXB2 reference sequence, converted to a numerical matrix, preprocessed using the preprocessing models prepared in the training phase. For each specimen, the corresponding HAND cluster was assigned by the multiclass random forest classifier trained during the clustering analysis. The original labels of neuropathological conditions and the prediction results were linked and visualized as a Sankey plot using the *googleVis* package [46].

### Estimation of the global burden of HAND using the Los Alamos HIV Sequence Database

The Los Alamos HIV Sequence Database (https://www.hiv.lanl.gov/content/sequence/HIV/mainpage.html) was used to demonstrate a retrospective estimation of the global burden of HAND. The sequences whose “culture method” were either “primary” or “uncultured” were downloaded. A total of 19800 *env* sequences from 1619 specimens were obtained. HAND status was predicted as described above.

## Additional files

**Additional file 1.**

**Additional file 2.**

## Abbreviations

HIV: human immunodeficiency virus type 1
HAND: HIV-associated neurocognitive disorder
cART: combination antiretroviral therapy
CNS: the central nervous system
HNRC: HIV Neurobehavioral Research Center
ANI: asymptomatic neurocognitive impairment
MND: mild neurocognitive disorder
HAD: HIV-associated dementia
ML: machine learning
CV: cross-validation
HIVE: HIV-associated encephalitis
NPD: non-specific neuropsychiatric disorder
FDR: false discovery rate
CSF: cerebrospinal fluid
AIDS: acquired immunodeficiency syndrome
NAb: neutralizing antibody
ADC: AIDS-dementia complex
SVM: support vector machine
RF: random forest
GBM: gradient boosting machine
XGBL: extreme gradient boosting with linear booster
XGBT: extreme gradient boosting with tree booster
CI: confidential interval
