# Supplementary material for: Prediction of HIV-associated neurocognitive disorder (HAND) from three genetic features of envelope gp120 glycoprotein
Source: Retrovirology. 2018 Jan 27;15:12. doi: 10.1186/s12977-018-0401-x (PMC5787250; doi:10.1186/s12977-018-0401-x)
Supplement: Supplementary file 1 — Additional file 1. [file 12977_2018_401_MOESM1_ESM.docx]

**Additional Files**


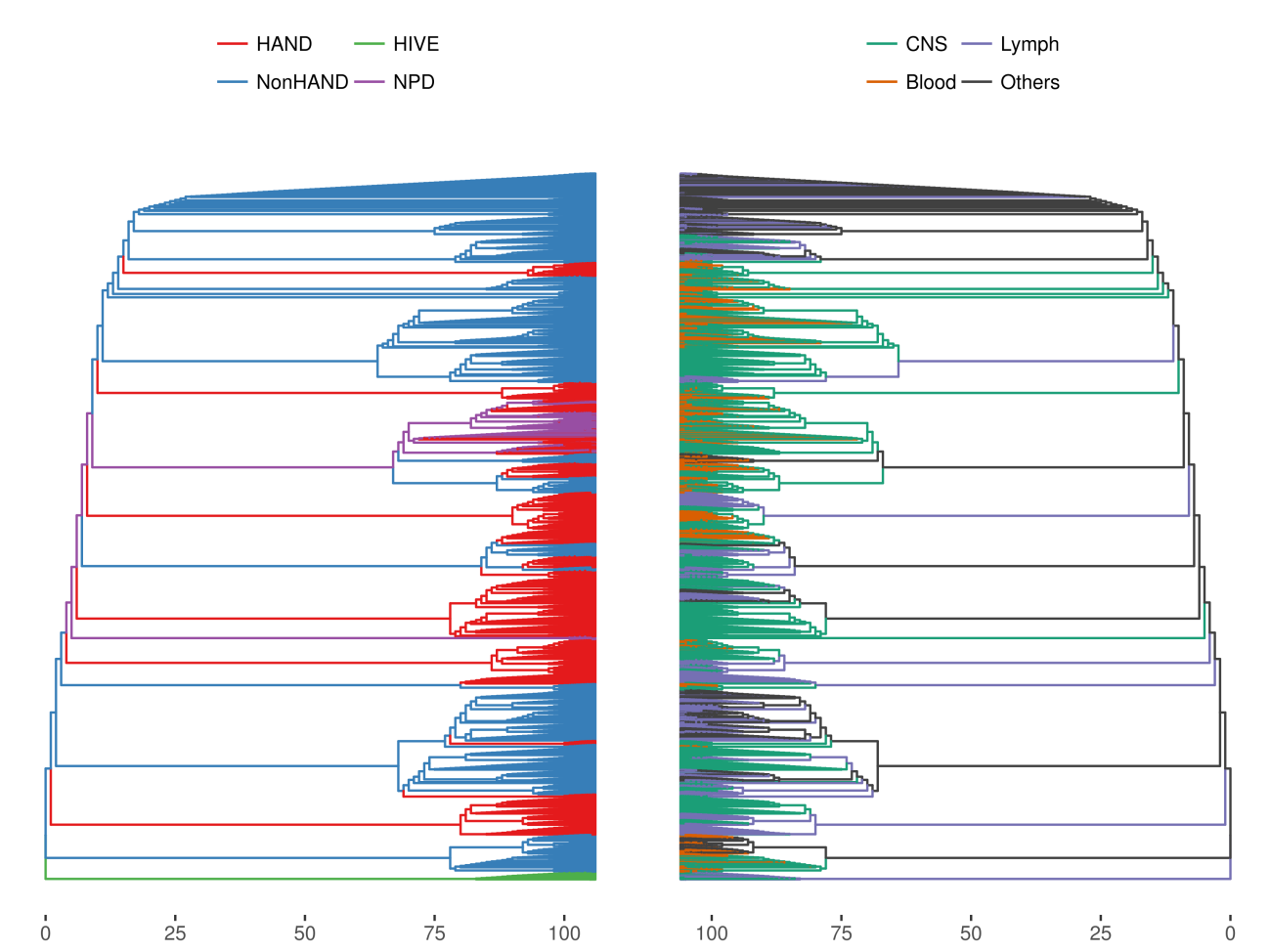


**Additional File 1: Figure 1. Phylogenetic analysis of the *env* C2V3C3 amino acid sequences.** The C2V3C3 regions were aligned and translated using the HIVAlign tool (<https://www.hiv.lanl.gov/content/sequence/VIRALIGN/viralign.html>). A phylogenetic tree was constructed from the amino acid alignment using Geneious software (<http://www.geneious.com/>). The built-in Geneious tree constructer was used with the default parameters not changed. Tree was then visualized by using the *ggtree* packages[47]. The left tree is colored on the basis of the neurocognitive status of the source patients. The right tree is colored on the basis of the sampling tissues.


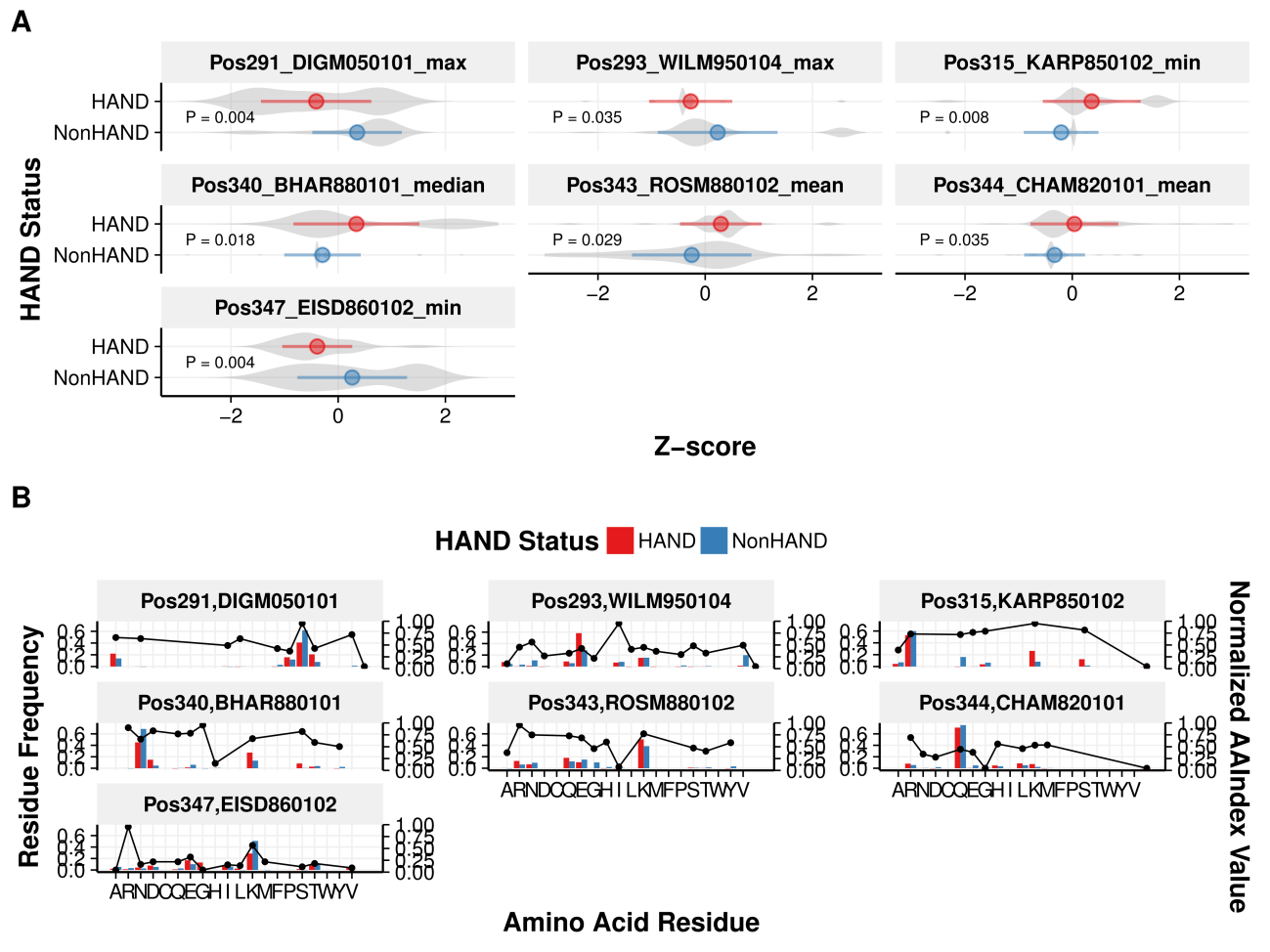


**Additional File 1: Figure 2. The set of features predictive of HAND obtained from feature importance analysis.** Model-specific feature importance was estimated using the *varImp* function implemented in the *caret* package for each of the ML algorithms except SVM. Features listed in the top 20 in two or more algorithms were selected. *P*-values were calculated using Welch’s *t*-test and adjusted by the FDR-based method[22]. Adjusted *P*-values of less than 0.05 were considered significant. In this manner, seven genetic features were retained (**Additional File 1: Figure 2**). (A) Distributions of detected features among HAND and NonHAND groups. The values of each feature were converted to Z-score for visualization purposes. (B) Scaled AAIndex values and relative residue frequencies in sequence sets derived from HAND and NonHAND cases. The weights of individual sequences are normalized by the respective sequencing depths of individual patients. The alignment position numbers correspond to the positions in the HXB2 HIV-1 sequence (accession: K03455).


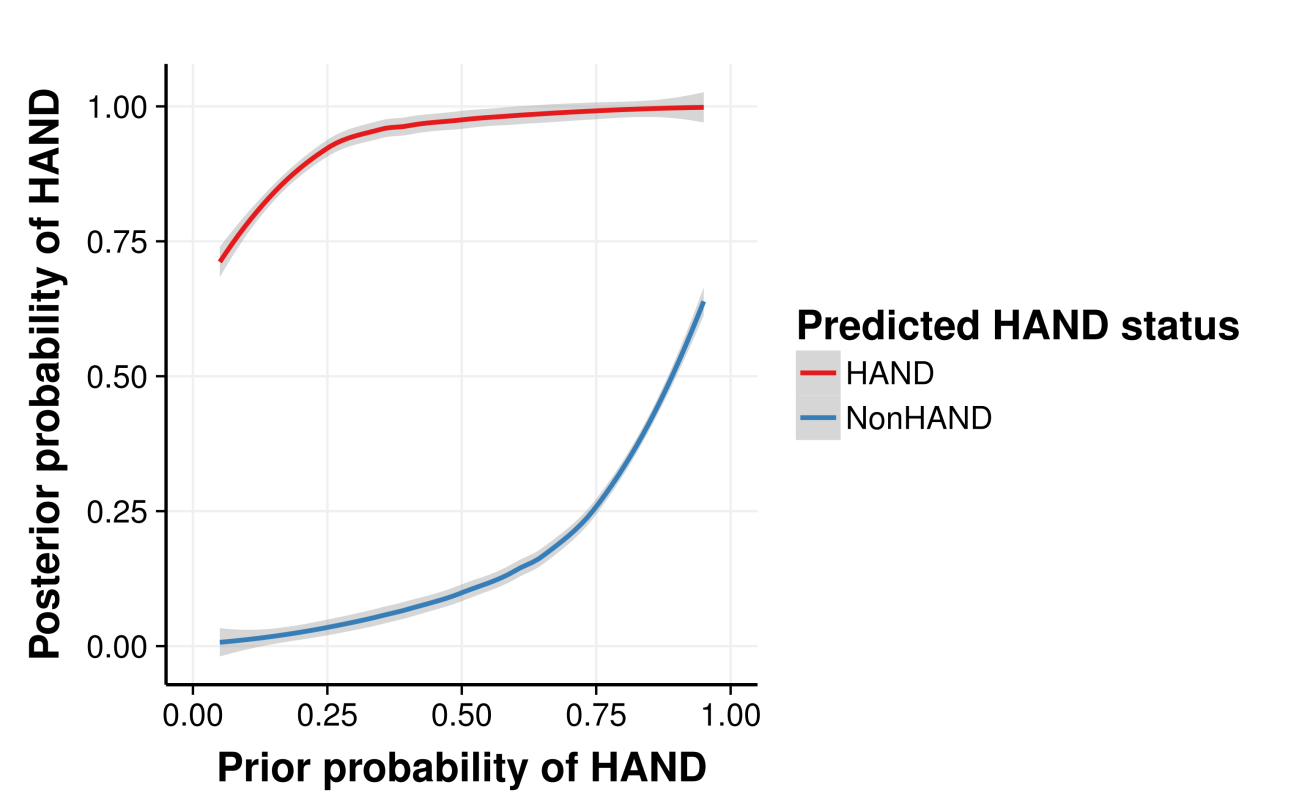


**Additional File 1: Figure 3. Distributions of the Bayesian posterior probabilities of HAND.** Different prior probabilities of HAND were tested to calculate the Bayesian posterior probabilities using the best stacked classifier (**Figure 1B**).


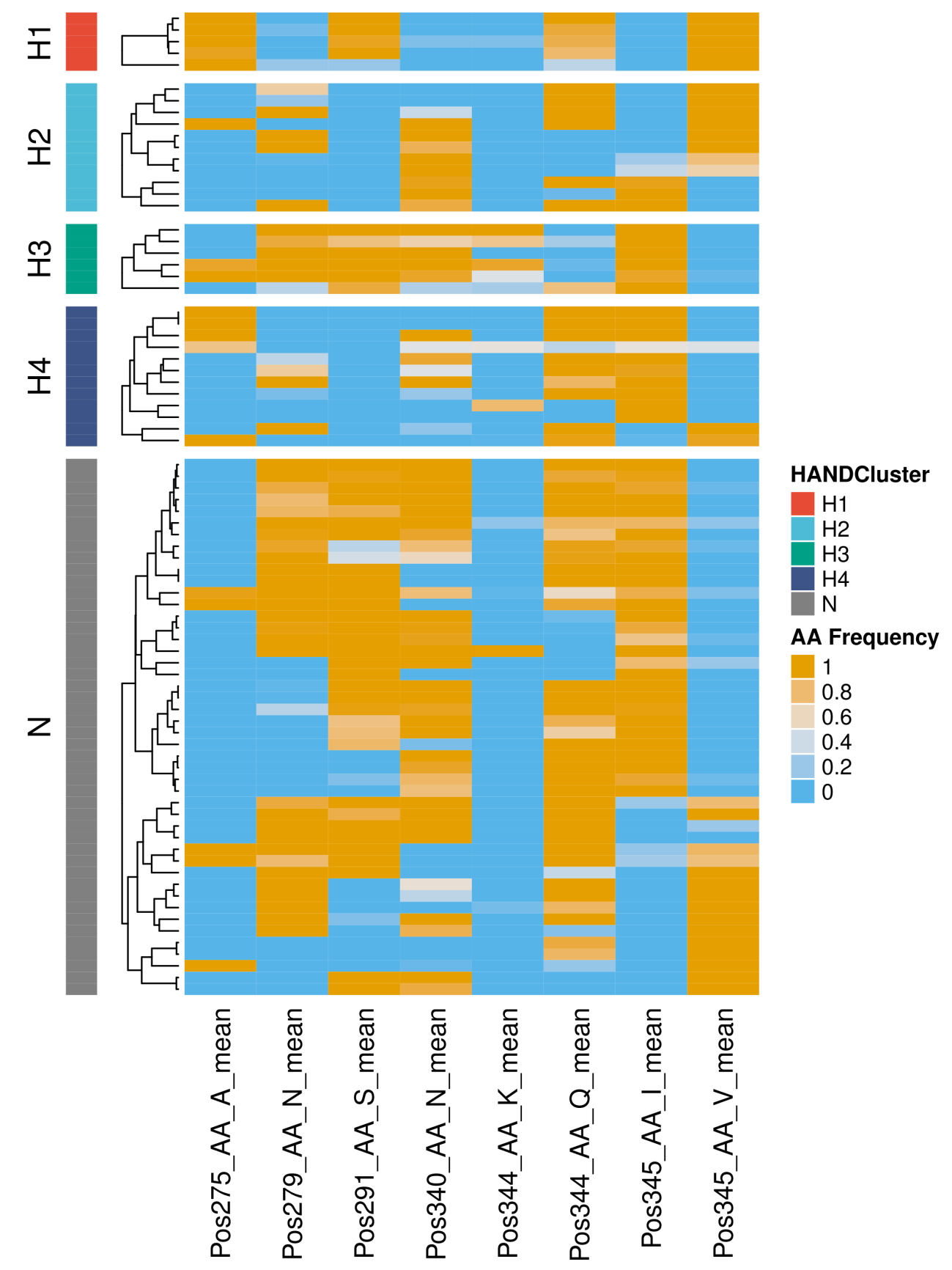


**Additional File 1: Figure 4. Amino acid variants varied among the HAND clusters.** The relative frequency of each variant was calculated, and the *preProcess* function in the *caret* package was used to screen out variants with no or little variance. HAND clusters were assigned using the multiclass RF classifier (**Figure 3B**). Feature importance was calculated using the filter approach implemented as the *filterVarImp* function in the *caret* package. Eight features whose importance values exceeded 0.9 for at least one class were selected and visualized.


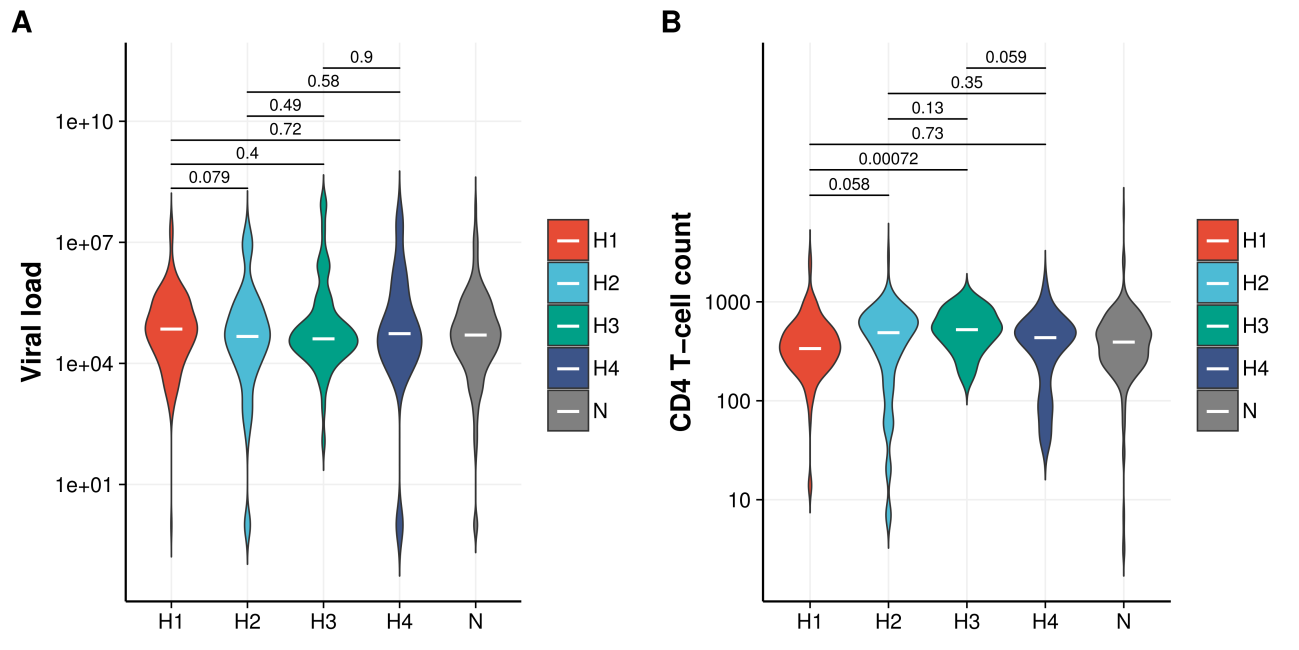


**Additional File 1: Figure 5. Distribution of HIV-specific parameters.** Annotation data retrieved from the Los Alamos HIV Sequence Database were depicted. Unadjusted *P*-values were calculated using the *wilcox.test* function, which performs Wilcoxon’s rank sum test. Bar represents the median value. For detailed information of the source dataset, see the legend of **Figure 5**.

**Additional File 1: Table 1. Characteristics of the sequence metadataset.**

|  | **HAND** | **NonHAND** | **HIVE** | **NPD** | ***P*-value** |
| --- | --- | --- | --- | --- | --- |
| N | 1015 | 1334 | 28 | 117 |  |
| # of unique specimens | 37 | 53 | 2 | 3 |  |
| # of unique patients | 19 | 21 | 1 | 2 |  |
| Sex (%) | | | | | <0.001 |
| Male | 393 (38.7) | 836 (62.7) | 0 ( 0.0) | 10 ( 8.5) |  |
| Female | 58 (5.7) | 87 (6.5) | 0 ( 0.0) | 0 ( 0.0) |  |
| Unknown | 564 (55.6) | 411 (30.8) | 28 (100.0) | 107 ( 91.5) |  |
| Georegion (%) | | | | | <0.001 |
| Europe | 27 ( 2.7) | 76 ( 5.7) | 0 ( 0.0) | 0 ( 0.0) |  |
| North America | 988 (97.3) | 1191 (89.3) | 28 (100.0) | 117 (100.0) |  |
| Sub-Saharan Africa | 0 ( 0.0) | 67 ( 5.0) | 0 ( 0.0) | 0 ( 0.0) |  |
| Sampling tissue (%) | | | | | <0.001 |
| Blood | 223 (22.0) | 291 (21.8) | 0 ( 0.0) | 57 ( 48.7) |  |
| CNS | 565 (55.7) | 436 (32.7) | 18 ( 64.3) | 60 ( 51.3) |  |
| Lymph | 203 (20.0) | 291 (21.8) | 10 ( 35.7) | 0 ( 0.0) |  |
| Others | 24 ( 2.4) | 316 (23.7) | 0 ( 0.0) | 0 ( 0.0) |  |
| Viral load (10^4^ copies) | 6.8 [1.7, 30.6] | 9.3 [7.2, 13.2] | NA | 4.8 [4.8, 5.8] | <0.001^†^ |
| CD4^+^ T-cell count (/μl) | 66 [50, 173] | 267 [215, 324] | 7 [7, 7] | 145 [108, 145] | <0.001^†^ |

†: Non-parametric hypothesis testing.

HAND, HIV-associated neurocognitive disorder.

HIVE, HIV encephalitis.

NPD, Non-specific neuropsychiatric disorder.

**Additional File 1: Table 2. Machine learning with all features.**

ML was repeated ten times with different random seeds, and the mean and 95% confidential intervals (CIs) were calculated for each of the statistics.

| Algorithm | Stat | Mean | 95%CI [Lo] | 95%CI [Up] |
| --- | --- | --- | --- | --- |
| SVM | Accuracy | 0.650234 | 0.561616 | 0.738853 |
|  | AccuracyLower | 0.363404 | 0.283297 | 0.443511 |
|  | AccuracyUpper | 0.871376 | 0.812284 | 0.930468 |
|  | Sensitivity | 0.460847 | 0.292852 | 0.628841 |
|  | Specificity | 0.781966 | 0.62421 | 0.939723 |
| RF | Accuracy | 0.631156 | 0.489818 | 0.772493 |
|  | AccuracyLower | 0.355222 | 0.241409 | 0.469036 |
|  | AccuracyUpper | 0.851028 | 0.744922 | 0.957134 |
|  | Sensitivity | 0.596825 | 0.397397 | 0.796254 |
|  | Specificity | 0.655423 | 0.440933 | 0.869914 |
| GBM | Accuracy | 0.534234 | 0.404336 | 0.664132 |
|  | AccuracyLower | 0.269986 | 0.154987 | 0.384986 |
|  | AccuracyUpper | 0.78514 | 0.695653 | 0.874627 |
|  | Sensitivity | 0.491534 | 0.247918 | 0.735151 |
|  | Specificity | 0.550705 | 0.320847 | 0.780564 |
| XGBL | Accuracy | 0.465766 | 0.381213 | 0.550319 |
|  | AccuracyLower | 0.208909 | 0.138482 | 0.279336 |
|  | AccuracyUpper | 0.738332 | 0.673409 | 0.803255 |
|  | Sensitivity | 0.444974 | 0.276697 | 0.61325 |
|  | Specificity | 0.473545 | 0.310418 | 0.636672 |
| XGBT | Accuracy | 0.491031 | 0.373681 | 0.608381 |
|  | AccuracyLower | 0.233039 | 0.141843 | 0.324235 |
|  | AccuracyUpper | 0.753311 | 0.663101 | 0.84352 |
|  | Sensitivity | 0.443915 | 0.290151 | 0.597679 |
|  | Specificity | 0.514771 | 0.335695 | 0.693846 |
| Stack | Accuracy | 0.632295 | 0.542991 | 0.7216 |
|  | AccuracyLower | 0.34739 | 0.264805 | 0.429974 |
|  | AccuracyUpper | 0.860212 | 0.801258 | 0.919167 |
|  | Sensitivity | 0.352381 | 0.178583 | 0.526179 |
|  | Specificity | 0.832892 | 0.680927 | 0.984857 |

AccuracyLower, the lower 95% CI estimated from the internal cross validation.

AccuracyUpper, the upper 95% CI estimated from the internal cross validation.

**Additional File 1: Table 3. Feature importance analysis.**

Model-specific feature importance was estimated for each of the algorithms tested, except SVM. Twenty features were selected for each of the algorithms.

| **Position** | **Algorithm** | **AAIndex** | **Stat** | **Importance** |
| --- | --- | --- | --- | --- |
| Pos230 | XGBL | EISD860103 | median | 24.199694 |
|  | XGBT | ROSM880103 | median | 7.415906 |
| Pos238 | GBM | CHAM820101 | max | 20.481341 |
|  | XGBL | PONP800106 | max | 9.888734 |
|  |  | WILM950104 | mean | 8.749949 |
| Pos240 | RF | NADH010101 | mean | 65.483226 |
|  |  | KUMS000101 | mean | 61.636554 |
|  | GBM | KARP850102 | sd | 31.754403 |
| Pos275 | GBM | NADH010107 | mean | 17.298871 |
| Pos278 | XGBL | WILM950103 | mean | 5.592014 |
|  | XGBT | WILM950101 | min | 38.270125 |
| Pos283 | RF | BHAR880101 | mean | 92.516316 |
|  | GBM | DIGM050101 | max | 26.565846 |
|  |  | BHAR880101 | mean | 17.622156 |
|  | XGBL | JUKT750101 | median | 7.210222 |
|  | XGBT | BHAR880101 | mean | 44.006474 |
|  |  | NADH010107 | min | 15.016870 |
| Pos291 | RF | NADH010107 | min | 100.000000 |
|  |  | KARP850103 | max | 71.236982 |
|  | GBM | DIGM050101 | max | 13.682474 |
|  | XGBL | EISD860102 | min | 18.188603 |
|  |  | CIDH920103 | min | 6.964961 |
|  |  | DIGM050101 | max | 6.221055 |
|  |  | KARP850103 | mean | 5.867549 |
|  | XGBT | DIGM050101 | max | 55.083296 |
|  |  | CIDH920103 | min | 17.620212 |
| Pos293 | RF | EISD860102 | median | 67.796911 |
|  |  | WILM950104 | max | 65.328705 |
|  | GBM | WILM950104 | max | 100.000000 |
|  |  | PONP800105 | max | 24.992993 |
|  | XGBL | WILM950104 | max | 100.000000 |
|  |  | JUKT750101 | mean | 9.100064 |
|  | XGBT | WILM950104 | max | 100.000000 |
| Pos308 | XGBL | EISD860102 | mean | 17.032099 |
| Pos315 | RF | PONP800105 | min | 80.116969 |
|  |  | GOLD730101 | sd | 73.588826 |
|  |  | KUMS000102 | median | 70.824574 |
|  |  | CIDH920103 | min | 70.435848 |
|  |  | VINM940102 | min | 66.765231 |
|  |  | KARP850102 | min | 62.714236 |
|  | GBM | PONP800105 | max | 16.800948 |
|  | XGBL | KARP850102 | min | 46.674936 |
|  |  | NADH010107 | sd | 5.366043 |
|  | XGBT | KARP850102 | min | 32.708949 |
| Pos321G | XGBL | ZIMJ680101 | mean | 4.494208 |
| Pos335 | XGBT | CIDH920104 | mean | 11.616460 |
| Pos336 | XGBT | PONP800104 | median | 17.367094 |
| Pos337 | RF | EISD860102 | median | 83.300646 |
| Pos340 | RF | BHAR880101 | median | 67.393663 |
|  | GBM | WILM950102 | mean | 24.492747 |
|  |  | CIDH920101 | median | 14.110663 |
|  | XGBL | BHAR880101 | median | 36.706529 |
|  |  | WILM950102 | mean | 4.553011 |
|  | XGBT | CHAM830108 | median | 24.392199 |
|  |  | BHAR880101 | median | 11.661736 |
| Pos343 | RF | ROSM880102 | mean | 68.109792 |
|  | GBM | ROSM880101 | min | 24.310313 |
|  |  | VINM940103 | min | 23.455081 |
|  | XGBT | VINM940103 | min | 21.551791 |
|  |  | WILM950104 | max | 18.378542 |
|  |  | ROSM880102 | mean | 13.045719 |
|  |  | MANP780101 | min | 12.018780 |
| Pos344 | GBM | CHAM820101 | mean | 31.706419 |
|  | XGBT | CHAM820101 | mean | 25.130726 |
| Pos347 | RF | BHAR880101 | min | 95.296194 |
|  |  | EISD860102 | min | 75.816122 |
|  |  | MANP780101 | median | 66.241908 |
|  |  | PONP800105 | max | 65.981126 |
|  | GBM | KUMS000102 | min | 27.320525 |
|  |  | PONP800106 | median | 19.569783 |
|  |  | KUMS000102 | sd | 18.131294 |
|  |  | EISD860102 | min | 17.238343 |
|  | XGBT | JUKT750101 | min | 13.611059 |
| Pos354 | XGBL | CIDH920103 | min | 72.505181 |
|  | XGBT | WILM950104 | min | 60.992817 |
|  |  | CIDH920103 | min | 11.814251 |
| Pos360 | GBM | WILM950103 | sd | 35.620374 |
| Pos362 | XGBL | DIGM050101 | max | 9.770891 |
| Pos363 | GBM | CIDH920102 | min | 17.725763 |
|  | XGBL | PONP800104 | mean | 4.733883 |

**Additional File 1: Table 4. Stepwise feature reduction.**

ML procedures were iterated with one feature removed at a time. In each iteration, mean accuracy was calculated from the results from ten different random seeds. In each iteration, the least important feature, determined by the highest accuracy of the stacked classifier, was removed.

| StepwiseID | Algorithm | RemovedFeature | MeanAccuracy |
| --- | --- | --- | --- |
| 0 | SVM | NA | 0.655739 |
| 0 | RF | NA | 0.689779 |
| 0 | GBM | NA | 0.649634 |
| 0 | XGBL | NA | 0.687943 |
| 0 | XGBT | NA | 0.680469 |
| 0 | Stack | NA | 0.70466 |
| 1 | SVM | Pos291_AAIndex_DIGM050101_max | 0.673591 |
| 1 | SVM | Pos293_AAIndex_WILM950104_max | 0.64185 |
| 1 | SVM | Pos315_AAIndex_KARP850102_min | 0.681232 |
| 1 | SVM | Pos340_AAIndex_BHAR880101_median | 0.617959 |
| 1 | SVM | Pos343_AAIndex_ROSM880102_mean | 0.68168 |
| 1 | SVM | Pos344_AAIndex_CHAM820101_mean | 0.656202 |
| 1 | SVM | Pos347_AAIndex_EISD860102_min | 0.647573 |
| 1 | RF | Pos291_AAIndex_DIGM050101_max | 0.638411 |
| 1 | RF | Pos293_AAIndex_WILM950104_max | 0.727707 |
| 1 | RF | Pos315_AAIndex_KARP850102_min | 0.685582 |
| 1 | RF | Pos340_AAIndex_BHAR880101_median | 0.674206 |
| 1 | RF | Pos343_AAIndex_ROSM880102_mean | 0.678256 |
| 1 | RF | Pos344_AAIndex_CHAM820101_mean | 0.720533 |
| 1 | RF | Pos347_AAIndex_EISD860102_min | 0.706115 |
| 1 | GBM | Pos291_AAIndex_DIGM050101_max | 0.683516 |
| 1 | GBM | Pos293_AAIndex_WILM950104_max | 0.643218 |
| 1 | GBM | Pos315_AAIndex_KARP850102_min | 0.649023 |
| 1 | GBM | Pos340_AAIndex_BHAR880101_median | 0.663757 |
| 1 | GBM | Pos343_AAIndex_ROSM880102_mean | 0.633598 |
| 1 | GBM | Pos344_AAIndex_CHAM820101_mean | 0.702528 |
| 1 | GBM | Pos347_AAIndex_EISD860102_min | 0.659158 |
| 1 | XGBL | Pos291_AAIndex_DIGM050101_max | 0.67733 |
| 1 | XGBL | Pos293_AAIndex_WILM950104_max | 0.720762 |
| 1 | XGBL | Pos315_AAIndex_KARP850102_min | 0.624985 |
| 1 | XGBL | Pos340_AAIndex_BHAR880101_median | 0.677035 |
| 1 | XGBL | Pos343_AAIndex_ROSM880102_mean | 0.630937 |
| 1 | XGBL | Pos344_AAIndex_CHAM820101_mean | 0.698026 |
| 1 | XGBL | Pos347_AAIndex_EISD860102_min | 0.683511 |
| 1 | XGBT | Pos291_AAIndex_DIGM050101_max | 0.663212 |
| 1 | XGBT | Pos293_AAIndex_WILM950104_max | 0.661162 |
| 1 | XGBT | Pos315_AAIndex_KARP850102_min | 0.602086 |
| 1 | XGBT | Pos340_AAIndex_BHAR880101_median | 0.654747 |
| 1 | XGBT | Pos343_AAIndex_ROSM880102_mean | 0.680388 |
| 1 | XGBT | Pos344_AAIndex_CHAM820101_mean | 0.720762 |
| 1 | XGBT | Pos347_AAIndex_EISD860102_min | 0.720844 |
| 1 | Stack | Pos291_AAIndex_DIGM050101_max | 0.660236 |
| 1 | Stack | Pos293_AAIndex_WILM950104_max | 0.751135 |
| 1 | Stack | Pos315_AAIndex_KARP850102_min | 0.661772 |
| 1 | Stack | Pos340_AAIndex_BHAR880101_median | 0.682143 |
| 1 | Stack | Pos343_AAIndex_ROSM880102_mean | 0.671312 |
| 1 | Stack | Pos344_AAIndex_CHAM820101_mean | 0.729462 |
| 1 | Stack | Pos347_AAIndex_EISD860102_min | 0.696724 |
| 2 | SVM | Pos291_AAIndex_DIGM050101_max | 0.602315 |
| 2 | SVM | Pos315_AAIndex_KARP850102_min | 0.656812 |
| 2 | SVM | Pos340_AAIndex_BHAR880101_median | 0.569119 |
| 2 | SVM | Pos343_AAIndex_ROSM880102_mean | 0.63109 |
| 2 | SVM | Pos344_AAIndex_CHAM820101_mean | 0.64185 |
| 2 | SVM | Pos347_AAIndex_EISD860102_min | 0.648184 |
| 2 | RF | Pos291_AAIndex_DIGM050101_max | 0.670386 |
| 2 | RF | Pos315_AAIndex_KARP850102_min | 0.623464 |
| 2 | RF | Pos340_AAIndex_BHAR880101_median | 0.662073 |
| 2 | RF | Pos343_AAIndex_ROSM880102_mean | 0.719623 |
| 2 | RF | Pos344_AAIndex_CHAM820101_mean | 0.735872 |
| 2 | RF | Pos347_AAIndex_EISD860102_min | 0.74358 |
| 2 | GBM | Pos291_AAIndex_DIGM050101_max | 0.69824 |
| 2 | GBM | Pos315_AAIndex_KARP850102_min | 0.610399 |
| 2 | GBM | Pos340_AAIndex_BHAR880101_median | 0.635134 |
| 2 | GBM | Pos343_AAIndex_ROSM880102_mean | 0.578805 |
| 2 | GBM | Pos344_AAIndex_CHAM820101_mean | 0.641997 |
| 2 | GBM | Pos347_AAIndex_EISD860102_min | 0.665044 |
| 2 | XGBL | Pos291_AAIndex_DIGM050101_max | 0.66397 |
| 2 | XGBL | Pos315_AAIndex_KARP850102_min | 0.581487 |
| 2 | XGBL | Pos340_AAIndex_BHAR880101_median | 0.670319 |
| 2 | XGBL | Pos343_AAIndex_ROSM880102_mean | 0.670849 |
| 2 | XGBL | Pos344_AAIndex_CHAM820101_mean | 0.720299 |
| 2 | XGBL | Pos347_AAIndex_EISD860102_min | 0.714031 |
| 2 | XGBT | Pos291_AAIndex_DIGM050101_max | 0.677411 |
| 2 | XGBT | Pos315_AAIndex_KARP850102_min | 0.604996 |
| 2 | XGBT | Pos340_AAIndex_BHAR880101_median | 0.654894 |
| 2 | XGBT | Pos343_AAIndex_ROSM880102_mean | 0.631548 |
| 2 | XGBT | Pos344_AAIndex_CHAM820101_mean | 0.75434 |
| 2 | XGBT | Pos347_AAIndex_EISD860102_min | 0.702905 |
| 2 | Stack | Pos291_AAIndex_DIGM050101_max | 0.662449 |
| 2 | Stack | Pos315_AAIndex_KARP850102_min | 0.646892 |
| 2 | Stack | Pos340_AAIndex_BHAR880101_median | 0.679167 |
| 2 | Stack | Pos343_AAIndex_ROSM880102_mean | 0.710613 |
| 2 | Stack | Pos344_AAIndex_CHAM820101_mean | 0.752737 |
| 2 | Stack | Pos347_AAIndex_EISD860102_min | 0.729375 |
| 3 | SVM | Pos291_AAIndex_DIGM050101_max | 0.618946 |
| 3 | SVM | Pos315_AAIndex_KARP850102_min | 0.634442 |
| 3 | SVM | Pos340_AAIndex_BHAR880101_median | 0.562785 |
| 3 | SVM | Pos343_AAIndex_ROSM880102_mean | 0.616209 |
| 3 | SVM | Pos347_AAIndex_EISD860102_min | 0.624145 |
| 3 | RF | Pos291_AAIndex_DIGM050101_max | 0.647568 |
| 3 | RF | Pos315_AAIndex_KARP850102_min | 0.594149 |
| 3 | RF | Pos340_AAIndex_BHAR880101_median | 0.658181 |
| 3 | RF | Pos343_AAIndex_ROSM880102_mean | 0.758923 |
| 3 | RF | Pos347_AAIndex_EISD860102_min | 0.799975 |
| 3 | GBM | Pos291_AAIndex_DIGM050101_max | 0.664367 |
| 3 | GBM | Pos315_AAIndex_KARP850102_min | 0.634524 |
| 3 | GBM | Pos340_AAIndex_BHAR880101_median | 0.602086 |
| 3 | GBM | Pos343_AAIndex_ROSM880102_mean | 0.569037 |
| 3 | GBM | Pos347_AAIndex_EISD860102_min | 0.699084 |
| 3 | XGBL | Pos291_AAIndex_DIGM050101_max | 0.694424 |
| 3 | XGBL | Pos315_AAIndex_KARP850102_min | 0.561711 |
| 3 | XGBL | Pos340_AAIndex_BHAR880101_median | 0.662383 |
| 3 | XGBL | Pos343_AAIndex_ROSM880102_mean | 0.696642 |
| 3 | XGBL | Pos347_AAIndex_EISD860102_min | 0.776018 |
| 3 | XGBT | Pos291_AAIndex_DIGM050101_max | 0.709239 |
| 3 | XGBT | Pos315_AAIndex_KARP850102_min | 0.560043 |
| 3 | XGBT | Pos340_AAIndex_BHAR880101_median | 0.696724 |
| 3 | XGBT | Pos343_AAIndex_ROSM880102_mean | 0.689316 |
| 3 | XGBT | Pos347_AAIndex_EISD860102_min | 0.753729 |
| 3 | Stack | Pos291_AAIndex_DIGM050101_max | 0.678404 |
| 3 | Stack | Pos315_AAIndex_KARP850102_min | 0.600483 |
| 3 | Stack | Pos340_AAIndex_BHAR880101_median | 0.65757 |
| 3 | Stack | Pos343_AAIndex_ROSM880102_mean | 0.701455 |
| 3 | Stack | Pos347_AAIndex_EISD860102_min | 0.824776 |
| 4 | SVM | Pos291_AAIndex_DIGM050101_max | 0.561711 |
| 4 | SVM | Pos315_AAIndex_KARP850102_min | 0.650168 |
| 4 | SVM | Pos340_AAIndex_BHAR880101_median | 0.553627 |
| 4 | SVM | Pos343_AAIndex_ROSM880102_mean | 0.614606 |
| 4 | RF | Pos291_AAIndex_DIGM050101_max | 0.710679 |
| 4 | RF | Pos315_AAIndex_KARP850102_min | 0.665359 |
| 4 | RF | Pos340_AAIndex_BHAR880101_median | 0.762276 |
| 4 | RF | Pos343_AAIndex_ROSM880102_mean | 0.813568 |
| 4 | GBM | Pos291_AAIndex_DIGM050101_max | 0.723799 |
| 4 | GBM | Pos315_AAIndex_KARP850102_min | 0.647273 |
| 4 | GBM | Pos340_AAIndex_BHAR880101_median | 0.624985 |
| 4 | GBM | Pos343_AAIndex_ROSM880102_mean | 0.578658 |
| 4 | XGBL | Pos291_AAIndex_DIGM050101_max | 0.745396 |
| 4 | XGBL | Pos315_AAIndex_KARP850102_min | 0.639337 |
| 4 | XGBL | Pos340_AAIndex_BHAR880101_median | 0.743432 |
| 4 | XGBL | Pos343_AAIndex_ROSM880102_mean | 0.765491 |
| 4 | XGBT | Pos291_AAIndex_DIGM050101_max | 0.697548 |
| 4 | XGBT | Pos315_AAIndex_KARP850102_min | 0.637734 |
| 4 | XGBT | Pos340_AAIndex_BHAR880101_median | 0.732443 |
| 4 | XGBT | Pos343_AAIndex_ROSM880102_mean | 0.752956 |
| 4 | Stack | Pos291_AAIndex_DIGM050101_max | 0.703882 |
| 4 | Stack | Pos315_AAIndex_KARP850102_min | 0.689169 |
| 4 | Stack | Pos340_AAIndex_BHAR880101_median | 0.745182 |
| 4 | Stack | Pos343_AAIndex_ROSM880102_mean | 0.756181 |
| 5 | SVM | Pos291_AAIndex_DIGM050101_max | 0.643137 |
| 5 | SVM | Pos315_AAIndex_KARP850102_min | 0.672456 |
| 5 | SVM | Pos340_AAIndex_BHAR880101_median | 0.513863 |
| 5 | RF | Pos291_AAIndex_DIGM050101_max | 0.798143 |
| 5 | RF | Pos315_AAIndex_KARP850102_min | 0.547227 |
| 5 | RF | Pos340_AAIndex_BHAR880101_median | 0.784865 |
| 5 | GBM | Pos291_AAIndex_DIGM050101_max | 0.672583 |
| 5 | GBM | Pos315_AAIndex_KARP850102_min | 0.602997 |
| 5 | GBM | Pos340_AAIndex_BHAR880101_median | 0.65726 |
| 5 | XGBL | Pos291_AAIndex_DIGM050101_max | 0.744785 |
| 5 | XGBL | Pos315_AAIndex_KARP850102_min | 0.601028 |
| 5 | XGBL | Pos340_AAIndex_BHAR880101_median | 0.79097 |
| 5 | XGBT | Pos291_AAIndex_DIGM050101_max | 0.760048 |
| 5 | XGBT | Pos315_AAIndex_KARP850102_min | 0.578047 |
| 5 | XGBT | Pos340_AAIndex_BHAR880101_median | 0.726786 |
| 5 | Stack | Pos291_AAIndex_DIGM050101_max | 0.775921 |
| 5 | Stack | Pos315_AAIndex_KARP850102_min | 0.617282 |
| 5 | Stack | Pos340_AAIndex_BHAR880101_median | 0.768992 |

**Additional File 1: Table 5. Machine learning with the optimized features.**

ML was repeated ten times with different random seeds, and the mean and 95% CIs were calculated for each of the statistics.

| Algorithm | Stat | Mean | 95%CI [Lo] | 95%CI [Up] |
| --- | --- | --- | --- | --- |
| SVM | Accuracy | 0.614606 | 0.481257 | 0.747955 |
|  | AccuracyLower | 0.339054 | 0.217409 | 0.460699 |
|  | AccuracyUpper | 0.841054 | 0.752932 | 0.929175 |
|  | Sensitivity | 0.739683 | 0.467093 | 1.012272 |
|  | Specificity | 0.514771 | 0.349084 | 0.680457 |
| RF | Accuracy | 0.813568 | 0.724002 | 0.903135 |
|  | AccuracyLower | 0.5323 | 0.423496 | 0.641103 |
|  | AccuracyUpper | 0.954803 | 0.919971 | 0.989634 |
|  | Sensitivity | 0.724868 | 0.575132 | 0.874603 |
|  | Specificity | 0.875 | 0.767575 | 0.982425 |
| GBM | Accuracy | 0.578658 | 0.510437 | 0.646879 |
|  | AccuracyLower | 0.297627 | 0.236949 | 0.358305 |
|  | AccuracyUpper | 0.825641 | 0.778166 | 0.873115 |
|  | Sensitivity | 0.468783 | 0.2837 | 0.653866 |
|  | Specificity | 0.651014 | 0.491434 | 0.810594 |
| XGBL | Accuracy | 0.765491 | 0.641457 | 0.889526 |
|  | AccuracyLower | 0.488692 | 0.344893 | 0.632491 |
|  | AccuracyUpper | 0.924231 | 0.873273 | 0.975189 |
|  | Sensitivity | 0.693122 | 0.544134 | 0.84211 |
|  | Specificity | 0.811508 | 0.647064 | 0.975952 |
| XGBT | Accuracy | 0.752956 | 0.630989 | 0.874923 |
|  | AccuracyLower | 0.47474 | 0.336064 | 0.613416 |
|  | AccuracyUpper | 0.917713 | 0.869417 | 0.966009 |
|  | Sensitivity | 0.737566 | 0.572644 | 0.902488 |
|  | Specificity | 0.762125 | 0.595886 | 0.928364 |
| Stack | Accuracy | 0.756181 | 0.576914 | 0.935449 |
|  | AccuracyLower | 0.493047 | 0.30718 | 0.678914 |
|  | AccuracyUpper | 0.90391 | 0.807485 | 1.000335 |
|  | Sensitivity | 0.732275 | 0.537608 | 0.926943 |
|  | Specificity | 0.767857 | 0.562307 | 0.973407 |

AccuracyLower, the lower 95% CI estimated from the internal cross validation.

AccuracyUpper, the upper 95% CI estimated from the internal cross validation.
