# Supplementary material for: Prediction of HIV-associated neurocognitive disorder (HAND) from three genetic features of envelope gp120 glycoprotein
Source: Retrovirology. 2018 Jan 27;15:12. doi: 10.1186/s12977-018-0401-x (PMC5787250; doi:10.1186/s12977-018-0401-x)
Supplement: Supplementary file 2 — Additional file 2. [file 12977_2018_401_MOESM2_ESM.html]

Molecular stratification of HAND from HIV env genetic signatures.


# Molecular stratification of HAND from HIV env genetic signatures.

#### *Masato Ogishi*

#### *2017/08/19 11:36*

- 1 Working environment
  - 1.1 Knitr options.
  - 1.2 R options.
  - 1.3 Data and packages.
- 2 Data
  - 2.1 Import & combine the sequence alignment and metadata.
  - 2.2 Create a summary table.
  - 2.3 Phylogenetic analysis.
- 3 Prediction of HAND status through machine learning
  - 3.1 Preparation of machine-learning-ready data.
  - 3.2 Machine learning options.
  - 3.3 Comparison of various classifiers trained with all features.
  - 3.4 The best stacked classifier trained with all features.
  - 3.5 Feature importance analysis.
  - 3.6 Elaborate the classifiers by stepwise feature reduction.
  - 3.7 Re-train the classifier with the minimal set of the most important features.
  - 3.8 The best stacked classifier with the optimized feature set.
  - 3.9 The most important features for the prediction of HAND status.
  - 3.10 Prediction summary.
  - 3.11 Baysian posterior probabilities.
  - 3.12 Predicting the HAND status for other neuropshychiatrically impaired cases.
- 4 Molecular stratification of HAND through the minimal set of genetic signatures
  - 4.1 Stratifying “HAND” by means of genetic clustering.
  - 4.2 Important amino acids for each of the HAND clusters.
  - 4.3 Characterize the HAND clusters by referencing the HAND database.
- 5 Estimating the global burden of HAND
  - 5.1 Los Alamos HIV Sequence Database.
  - 5.2 Global burden of HAND.
  - 5.3 Correlation of predicted HAND clusters and HIV-specific parameters.
- 6 Closing
  - 6.1 Session info.
  - 6.2 Save the workspace.

# 1 Working environment

## 1.1 Knitr options.

```
knitr::opts_chunk$set(results="hide")
knitr::opts_chunk$set(echo=T)
knitr::opts_chunk$set(eval=T)
knitr::opts_chunk$set(tidy=F)
knitr::opts_chunk$set(warning=F)
knitr::opts_chunk$set(error=F)
knitr::opts_chunk$set(message=F)
```

## 1.2 R options.

```
# Enable parallel computing
library(doParallel)
cl <- makePSOCKcluster(6)
registerDoParallel(cl)

# Avoid exponential expressions
options(scipen=100)
```

## 1.3 Data and packages.

```
## load(file="HANDPrediction_Workspace.RData")

# Packages
library(devtools)
load_all() # library(HANDPrediction)
library(tidyverse)
library(RColorBrewer)
library(ggsci)
library(googleVis)
library(caret)
```

# 2 Data

## 2.1 Import & combine the sequence alignment and metadata.

```
df.hand <- dataImportAndCombine(
  fileName.Alignment.FASTA=system.file("C2V3C3_Metadataset_AA.fasta", package="HANDPrediction"),
  fileName.HIVNeuroMetadata.CSV=system.file("Metadataset.csv", package="HANDPrediction"),
  fileName.SampleTissueCategoryDesignSheet.CSV=system.file(
    "Metadataset_SampleTissue_DesignSheet.csv", package="HANDPrediction"
    ),
  colName.SequenceID="Accession",
  colName.Sample.Tissue="Sample.Tissue",
  colName.Clinical.Status="Clinical.Status"
) %>%
  dplyr::filter(Clinical.Status %in% c("HAND", "NonHAND")) %>%
  dplyr::mutate(Clinical.Status=factor(Clinical.Status, levels=c("HAND", "NonHAND", "HIVE", "NPD")))
df.others <- dataImportAndCombine(
  fileName.Alignment.FASTA=system.file("C2V3C3_Metadataset_AA.fasta", package="HANDPrediction"),
  fileName.HIVNeuroMetadata.CSV=system.file("Metadataset.csv", package="HANDPrediction"),
  fileName.SampleTissueCategoryDesignSheet.CSV=system.file(
    "Metadataset_SampleTissue_DesignSheet.csv", package="HANDPrediction"
    ),
  colName.SequenceID="Accession",
  colName.Sample.Tissue="Sample.Tissue",
  colName.Clinical.Status="Clinical.Status"
) %>%
  dplyr::filter(Clinical.Status %in% c("HIVE", "NPD")) %>%
  dplyr::mutate(Clinical.Status=factor(Clinical.Status, levels=c("HAND", "NonHAND", "HIVE", "NPD")))
df.all <- dplyr::bind_rows(df.hand, df.others)
```

## 2.2 Create a summary table.

```
tableOne.hand <- combinedDatasetSummaryTable(
  combinedDataset=df.all, 
  strataColName="Clinical.Status",
  varColNamesString="Patient.Sex|Georegion|Sample.Tissue.Category|Viral.load|CD4.count|Number.of.patient.seqs",
  factorColNamesString="Patient.Sex|Georegion|Sample.Tissue.Category",
  nonNormalColNameString="Viral.load|CD4.count|Number.of.patient.seqs"
)
```

```
##                                        Stratified by Clinical.Status
##                                         HAND                          
##   n                                         1015                      
##   Patient.Sex (%)                                                     
##      F                                        58 ( 5.7)               
##      M                                       393 (38.7)               
##      NA                                      564 (55.6)               
##   Georegion (%)                                                       
##      Europe                                   27 ( 2.7)               
##      North America                           988 (97.3)               
##      Sub-Saharan Africa                        0 ( 0.0)               
##   Sample.Tissue.Category (%)                                          
##      Blood                                   223 (22.0)               
##      CNS                                     565 (55.7)               
##      Lymph                                   203 (20.0)               
##      Others                                   24 ( 2.4)               
##   Viral.load (median [IQR])             67608.00 [17378.00, 305777.00]
##   CD4.count (median [IQR])                 66.00 [50.00, 173.00]      
##   Number.of.patient.seqs (median [IQR])    66.00 [53.00, 196.00]      
##                                        Stratified by Clinical.Status
##                                         NonHAND                       
##   n                                         1334                      
##   Patient.Sex (%)                                                     
##      F                                        87 ( 6.5)               
##      M                                       836 (62.7)               
##      NA                                      411 (30.8)               
##   Georegion (%)                                                       
##      Europe                                   76 ( 5.7)               
##      North America                          1191 (89.3)               
##      Sub-Saharan Africa                       67 ( 5.0)               
##   Sample.Tissue.Category (%)                                          
##      Blood                                   291 (21.8)               
##      CNS                                     436 (32.7)               
##      Lymph                                   291 (21.8)               
##      Others                                  316 (23.7)               
##   Viral.load (median [IQR])             93325.00 [72444.00, 131826.00]
##   CD4.count (median [IQR])                267.00 [215.00, 324.00]     
##   Number.of.patient.seqs (median [IQR])   181.00 [65.00, 225.00]      
##                                        Stratified by Clinical.Status
##                                         HIVE                
##   n                                        28               
##   Patient.Sex (%)                                           
##      F                                      0 (  0.0)       
##      M                                      0 (  0.0)       
##      NA                                    28 (100.0)       
##   Georegion (%)                                             
##      Europe                                 0 (  0.0)       
##      North America                         28 (100.0)       
##      Sub-Saharan Africa                     0 (  0.0)       
##   Sample.Tissue.Category (%)                                
##      Blood                                  0 (  0.0)       
##      CNS                                   18 ( 64.3)       
##      Lymph                                 10 ( 35.7)       
##      Others                                 0 (  0.0)       
##   Viral.load (median [IQR])                NA [NA, NA]      
##   CD4.count (median [IQR])               7.00 [7.00, 7.00]  
##   Number.of.patient.seqs (median [IQR]) 28.00 [28.00, 28.00]
##                                        Stratified by Clinical.Status
##                                         NPD                          
##   n                                          117                     
##   Patient.Sex (%)                                                    
##      F                                         0 (  0.0)             
##      M                                        10 (  8.5)             
##      NA                                      107 ( 91.5)             
##   Georegion (%)                                                      
##      Europe                                    0 (  0.0)             
##      North America                           117 (100.0)             
##      Sub-Saharan Africa                        0 (  0.0)             
##   Sample.Tissue.Category (%)                                         
##      Blood                                    57 ( 48.7)             
##      CNS                                      60 ( 51.3)             
##      Lymph                                     0 (  0.0)             
##      Others                                    0 (  0.0)             
##   Viral.load (median [IQR])             47863.00 [47863.00, 57544.00]
##   CD4.count (median [IQR])                145.00 [108.00, 145.00]    
##   Number.of.patient.seqs (median [IQR])   196.00 [196.00, 196.00]    
##                                        Stratified by Clinical.Status
##                                         p      test   
##   n                                                   
##   Patient.Sex (%)                       <0.001        
##      F                                                
##      M                                                
##      NA                                               
##   Georegion (%)                         <0.001        
##      Europe                                           
##      North America                                    
##      Sub-Saharan Africa                               
##   Sample.Tissue.Category (%)            <0.001        
##      Blood                                            
##      CNS                                              
##      Lymph                                            
##      Others                                           
##   Viral.load (median [IQR])             <0.001 nonnorm
##   CD4.count (median [IQR])              <0.001 nonnorm
##   Number.of.patient.seqs (median [IQR]) <0.001 nonnorm
```

```
combinedDatasetSummaryTable.Export(
  tableOne.hand,
  outputFileName="./results/SupplementaryTable1.docx"
)
```

## 2.3 Phylogenetic analysis.

```
phylo_colors <- list(brewer.pal(4, "Set1"), c(brewer.pal(3, "Dark2"), "grey25"))
ggPhyloTree.Mirror(
  tree.newick.file=system.file("C2V3C3_Metadataset_AA_PhyloTree.newick", package="HANDPrediction"), 
  combinedDataset=df.all, 
  groupLabelNames=c("Clinical.Status", "Sample.Tissue.Category"),
  groupVarNamesList=list(c("HAND", "NonHAND", "HIVE", "NPD"), c("CNS", "Blood", "Lymph", "Others")),
  colorSetList=phylo_colors, tipped=F
)
```

```
saveCurrentGraphicPDF("./figures/SupplementaryFigure1.pdf", width=8, height=6)
```

# 3 Prediction of HAND status through machine learning

## 3.1 Preparation of machine-learning-ready data.

```
ML_Matrix <- machineLearning.Matrix(
  sequences=df.hand$"Sequence.AA", 
  analysisType="AAIndex", 
  aaIndexType="physicochemical", 
  normalized=T
) 
ML_Data <- machineLearning.DataFormat(
  ml.matrix=ML_Matrix,
  ml.metadata=df.hand,
  colNames.from=c("Reference_PMID","LANL_Patient.Id","Clinical.Status","Sample.Tissue.Category"), 
  colNames.to=c("StudyID","PatientID","NeuroOutcome","SampleTissue"), 
  outcomeLabelName="NeuroOutcome", 
  sourceLabelName="SampleTissue", 
  pp="internal"
)
```

## 3.2 Machine learning options.

```
ML_Algorithms <- list("svmRadial", "rf", "gbm", "xgbLinear", "xgbTree")
ML_AlgorithmLabels <- list("SVM", "RF", "GBM", "XGBL", "XGBT")
ML_Options <- trainControl(
  method="repeatedcv", number=3, repeats=10, savePredictions="final", verboseIter=F, classProbs=T
)
```

## 3.3 Comparison of various classifiers trained with all features.

```
ML_ALL_Bootstrap <- machineLearning.Comparison.Bootstrap(
  ml.data=ML_Data$"MLDataFrame", 
  ml.metadata=ML_Data$"MLMetadataDF", 
  holdoutTrainingRatio=0.8,
  outcomeLabelName="NeuroOutcome", 
  patientIDLabelName="PatientID",
  algorithmList=ML_Algorithms, 
  algorithmLabelList=ML_AlgorithmLabels, 
  trainControlOptions=ML_Options,
  seedList=c(1, 12, 123, 1234, 12345, 123456, 1234567, 12345678, 123456789)
)
write.csv(ML_ALL_Bootstrap$MLStatDF.Summary, file="./results/SupplementaryTable2.csv", row.names=F)
Fig.1A <- ML_ALL_Bootstrap$"AccuracyPlot"
```

## 3.4 The best stacked classifier trained with all features.

```
ML_ALL_BestSeed <- ML_ALL_Bootstrap$MLStatDF %>% dplyr::filter(Algorithm=="Stack") %>%
  DescTools::Sort(ord=c("Accuracy","AccuracyLower","AccuracyUpper"), decreasing=T) %>% (function(d){d[["RandomSeed"]][1]})
ML_ALL_Best <- machineLearning.Comparison(
  ml.data=ML_Data$"MLDataFrame", 
  ml.metadata=ML_Data$"MLMetadataDF", 
  holdoutTrainingRatio=0.8,
  outcomeLabelName="NeuroOutcome", 
  patientIDLabelName="PatientID",
  algorithmList=ML_Algorithms, 
  algorithmLabelList=ML_AlgorithmLabels, 
  trainControlOptions=ML_Options,
  seed=ML_ALL_BestSeed
)
```

## 3.5 Feature importance analysis.

```
ML_ImportantFeatures <- machineLearning.FeatureImportanceAnalysis(
  ml.data=ML_Data$"MLDataFrame",
  outcomeLabelName="NeuroOutcome",
  modelList=ML_ALL_Best$"MLModelList"[-length(ML_Algorithms)-1],
  outputFileName.DOCX="./results/SupplementaryTable3.docx",
  xLabel="HAND Status",
  yLabel="Z-score",
  colorSet=brewer.pal(3, "Set1")[2:1]
)
Fig.S2A <- ML_ImportantFeatures$"ViolinPlot"
Fig.S2B <- machineLearning.ResidueAAIndexDualPlot(
  featureSet=ML_ImportantFeatures$"MostImportantFeatures", 
  sequences=df.hand$"Sequence.AA", 
  seqWeights=df.hand$"Number.of.patient.seqs",
  outcomes=df.hand$"Clinical.Status", 
  xLabel="Amino Acid Residue", 
  yLabel1="Residue Frequency",
  yLabel2="Normalized AAIndex Value",
  legendLabel="HAND Status", 
  colorSet=brewer.pal(3, "Set1")[1:2]
)
Fig.S2 <- cowplot::plot_grid(Fig.S2A, Fig.S2B, labels="AUTO", label_size=20, ncol=1, align="none")
savePDF(Fig.S2, "./figures/SupplementaryFigure2.pdf", width=12, height=9)
```

## 3.6 Elaborate the classifiers by stepwise feature reduction.

```
ML_FeatureReduction <- machineLearning.StepwiseFeatureReduction(
  featureSet=ML_ImportantFeatures$"MostImportantFeatures",
  ml.data=ML_Data$"MLDataFrame",
  ml.metadata=ML_Data$"MLMetadataDF", 
  holdoutTrainingRatio=0.8,
  outcomeLabelName="NeuroOutcome", 
  patientIDLabelName="PatientID",
  algorithmList=ML_Algorithms, 
  algorithmLabelList=ML_AlgorithmLabels, 
  trainControlOptions=ML_Options,
  seedList=c(1, 12, 123, 1234, 12345, 123456, 1234567, 12345678, 123456789)
)
saveRDS(ML_FeatureReduction, "./results/StepwiseFeatureReduction.rds")
write.csv(ML_FeatureReduction$StepwiseStatSummaryDF, file="./results/SupplementaryTable4.csv", row.names=F)
```

## 3.7 Re-train the classifier with the minimal set of the most important features.

```
ML_Data$"MLDataFrame_Minimum" <- dplyr::select(
  ML_Data$"MLDataFrame", 
  dplyr::matches(paste0(c("NeuroOutcome", ML_FeatureReduction$"MostImportantFeatures"), collapse="|"))
) 
ML_Minimum_Bootstrap <- machineLearning.Comparison.Bootstrap(
  ml.data=ML_Data$"MLDataFrame_Minimum", 
  ml.metadata=ML_Data$"MLMetadataDF", 
  holdoutTrainingRatio=0.8,
  outcomeLabelName="NeuroOutcome", 
  patientIDLabelName="PatientID",
  algorithmList=ML_Algorithms, 
  algorithmLabelList=ML_AlgorithmLabels, 
  trainControlOptions=ML_Options,
  seedList=c(1, 12, 123, 1234, 12345, 123456, 1234567, 12345678, 123456789)
)
write.csv(ML_Minimum_Bootstrap$MLStatDF.Summary, file="./results/SupplementaryTable5.csv", row.names=F)
Fig.1B <- ML_Minimum_Bootstrap$"AccuracyPlot"
Fig.1 <- cowplot::plot_grid(Fig.1A, Fig.1B, labels="AUTO", label_size=20, nrow=1, align="none")
savePDF(Fig.1, "./figures/Figure1.pdf", w=10, h=5)
```

## 3.8 The best stacked classifier with the optimized feature set.

```
ML_Minimum_BestSeed <- ML_Minimum_Bootstrap$MLStatDF %>% dplyr::filter(Algorithm=="Stack") %>%
  DescTools::Sort(ord=c("Accuracy","AccuracyLower","AccuracyUpper"), decreasing=T) %>% (function(d){d[["RandomSeed"]][1]})
ML_Minimum_Best <- machineLearning.Comparison(
  ml.data=ML_Data$"MLDataFrame_Minimum", 
  ml.metadata=ML_Data$"MLMetadataDF", 
  holdoutTrainingRatio=0.8,
  outcomeLabelName="NeuroOutcome", 
  patientIDLabelName="PatientID",
  algorithmList=ML_Algorithms, 
  algorithmLabelList=ML_AlgorithmLabels, 
  trainControlOptions=ML_Options,
  seed=ML_Minimum_BestSeed
)
ML_Minimum_Best_Classifiers <- list()
ML_Minimum_Best_Classifiers$"MLModelList" <- ML_Minimum_Best$"MLModelList"[-length(ML_Algorithms)-1]
ML_Minimum_Best_Classifiers$"MLStackingModel" <- ML_Minimum_Best$"MLModelList"[[length(ML_Algorithms)+1]]
```

## 3.9 The most important features for the prediction of HAND status.

```
ML_Minimum_Features <- machineLearning.FeatureImportanceAnalysis(
  ml.data=ML_Data$"MLDataFrame_Minimum",
  outcomeLabelName="NeuroOutcome",
  modelList=ML_Minimum_Best$"MLModelList"[-length(ML_Algorithms)-1],
  outputFileName.DOCX=NULL,
  xLabel="HAND Status",
  yLabel="Z-score",
  colorSet=brewer.pal(3, "Set1")[2:1]
)
Fig.2A <- ML_Minimum_Features$"ViolinPlot"
Fig.2B <- machineLearning.ResidueAAIndexDualPlot(
  featureSet=ML_FeatureReduction$"MostImportantFeatures", 
  sequences=df.hand$"Sequence.AA", 
  seqWeights=df.hand$"Number.of.patient.seqs",
  outcomes=df.hand$"Clinical.Status", 
  xLabel="Amino Acid Residue", 
  yLabel1="Residue Frequency",
  yLabel2="Normalized AAIndex Value",
  legendLabel="HAND Status", 
  colorSet=brewer.pal(3, "Set1")[1:2]
)
Fig.2 <- cowplot::plot_grid(Fig.2A, Fig.2B, labels="AUTO", label_size=20, ncol=1, align="none")
savePDF(Fig.2, "./figures/Figure2.pdf", width=12, height=8)
```

## 3.10 Prediction summary.

```
ML_Minimum_Pred <- suppressWarnings(
  dplyr::bind_rows(
    dplyr::bind_cols(
      ML_Minimum_Best$"MLDataFrame_Training",
      machineLearning.Stacking.Predict(
        ML_Minimum_Best$"MLDataFrame_Training",
        modelList=ML_Minimum_Best_Classifiers$"MLModelList",
        model.stack=ML_Minimum_Best_Classifiers$"MLStackingModel"
      )
    ),
    dplyr::bind_cols(
      ML_Minimum_Best$"MLDataFrame_Testing",
      machineLearning.Stacking.Predict(
        ML_Minimum_Best$"MLDataFrame_Testing",
        modelList=ML_Minimum_Best_Classifiers$"MLModelList",
        model.stack=ML_Minimum_Best_Classifiers$"MLStackingModel"
      )
    )
  )
) %>% dplyr::mutate(Data=factor(Data, levels=c("Train", "Test")))
(ML_Minimum_Pred_CM <- caret::confusionMatrix(ML_Minimum_Pred$PredictedOutcome, ML_Minimum_Pred$NeuroOutcome))
```

```
## Confusion Matrix and Statistics
## 
##           Reference
## Prediction HAND NonHAND
##    HAND      33       1
##    NonHAND    4      42
##                                               
##                Accuracy : 0.9375              
##                  95% CI : (0.8601, 0.9794)    
##     No Information Rate : 0.5375              
##     P-Value [Acc > NIR] : 0.000000000000003302
##                                               
##                   Kappa : 0.8736              
##  Mcnemar's Test P-Value : 0.3711              
##                                               
##             Sensitivity : 0.8919              
##             Specificity : 0.9767              
##          Pos Pred Value : 0.9706              
##          Neg Pred Value : 0.9130              
##              Prevalence : 0.4625              
##          Detection Rate : 0.4125              
##    Detection Prevalence : 0.4250              
##       Balanced Accuracy : 0.9343              
##                                               
##        'Positive' Class : HAND                
##
```

## 3.11 Baysian posterior probabilities.

```
Fig.S3 <- bayesianPosteriorProbabilityPlot(confMat=ML_Minimum_Pred_CM)
savePDF(Fig.S3, "./figures/SupplementaryFigure3.pdf", width=8, height=5)
```

## 3.12 Predicting the HAND status for other neuropshychiatrically impaired cases.

```
df.others.pred <- machineLearning.Stacking.Predict(
  df.others$"Sequence.AA",
  ml.metadata=df.others,
  colNames.from=c("Reference_PMID","LANL_Patient.Id","Clinical.Status","Sample.Tissue.Category"), 
  colNames.to=c("StudyID","PatientID","NeuroOutcome","SampleTissue"), 
  outcomeLabelName=NULL, sourceLabelName="SampleTissue",
  pp="external", 
  pp.by.sequence=ML_Data$"MLDataPreProcessing_BySequence", 
  pp.by.patient=ML_Data$"MLDataPreProcessing_ByPatient",
  modelList=ML_Minimum_Best_Classifiers$"MLModelList",
  model.stack=ML_Minimum_Best_Classifiers$"MLStackingModel"
)
df.others.pred$"MLPredDF"
```

```
##    StudyID PatientID NeuroOutcome SampleTissue PredictedOutcome  SVM.HAND
## 1 10381169       887          NPD          CNS             HAND 0.7736563
## 2 22007152     47553          NPD        Blood             HAND 0.7735976
## 3 22007152     47553          NPD          CNS             HAND 0.7735976
## 4 22420378     47450         HIVE          CNS             HAND 0.7296019
## 5 22420378     47450         HIVE        Lymph             HAND 0.7555908
##   SVM.NonHAND RF.HAND RF.NonHAND  GBM.HAND GBM.NonHAND XGBL.HAND
## 1   0.2263437   0.988      0.012 0.4820161   0.5179839 0.9076127
## 2   0.2264024   0.976      0.024 0.6407601   0.3592399 0.9312313
## 3   0.2264024   0.976      0.024 0.6407601   0.3592399 0.9312313
## 4   0.2703981   0.982      0.018 0.8149778   0.1850222 0.9790089
## 5   0.2444092   0.998      0.002 0.8149778   0.1850222 0.9790089
##   XGBL.NonHAND XGBT.HAND XGBT.NonHAND Stack.HAND Stack.NonHAND
## 1   0.09238726 0.8820459  0.117954075  0.9713653    0.02863467
## 2   0.06876868 0.8623496  0.137650430  0.9713653    0.02863467
## 3   0.06876868 0.8623496  0.137650430  0.9713653    0.02863467
## 4   0.02099115 0.9772738  0.022726238  0.9713653    0.02863467
## 5   0.02099115 0.9913781  0.008621931  0.9713653    0.02863467
```

# 4 Molecular stratification of HAND through the minimal set of genetic signatures

## 4.1 Stratifying “HAND” by means of genetic clustering.

```
# Identify clusters from heatmap
set.seed(1)
library(ComplexHeatmap)
hm <- Heatmap(
  as.matrix(as.data.frame(lapply(
      dplyr::select(ML_Minimum_Pred, dplyr::contains("AAIndex"), dplyr::contains(".HAND")), 
      scales::rescale
    ))), 
  name="Feature",
  col=circlize::colorRamp2(c(0, 0.5, 1), c("#56B4E9", "grey90", "#E69F00")),
  row_dend_reorder=T, 
  cluster_columns=F,
  show_column_dend=F,
  km=8,
  km_title="C%i",
  clustering_distance_rows="pearson",
  gap=unit(2, "mm")
)
ha <- rowAnnotation(
  df=dplyr::select(ML_Minimum_Pred, Data, NeuroOutcome, PredictedOutcome, SampleTissue),
  col=list(Data=c("Train"="grey10", "Test"="grey90"),
           NeuroOutcome=c("HAND"=brewer.pal(3, "Set1")[1], 
                          "NonHAND"=brewer.pal(3, "Set1")[2]),
           PredictedOutcome=c("HAND"=paste0(brewer.pal(3, "Set1")[1],"90"), 
                              "NonHAND"=paste0(brewer.pal(3, "Set1")[2],"90")),
           SampleTissue=c("Blood"=brewer.pal(3, "Dark2")[1], 
                          "CNS"=brewer.pal(3, "Dark2")[2], 
                          "Lymph"=brewer.pal(3, "Dark2")[3],
                          "Others"="grey50"))
)
draw(ha + hm, padding=unit(c(40, 2, 2, 2), "mm"), row_dend_side="left", row_sub_title_side="right")
```

```
saveCurrentGraphicPDF("./figures/Figure3A.pdf", width=6, height=8)

# Manually determine HAND-enriched clusters.
set.seed(1) ## It seems necessary to repeat th edeclaration once again...
hm.clusters <- row_order(hm)
names(hm.clusters) <- paste0("C", 1:length(hm.clusters))
ML_Cluster <- ML_Minimum_Pred %>% dplyr::mutate(Cluster=0)
for(i in 1:length(hm.clusters)){
  ML_Cluster[["Cluster"]][hm.clusters[[i]]] <- names(hm.clusters)[i]
}
ML_Cluster <- ML_Cluster %>% dplyr::select(Data, StudyID, PatientID, NeuroOutcome, Cluster, dplyr::matches("AAIndex"))
hm.clusters <- ML_Cluster[["Cluster"]]
hm.clusters[grep("C2", hm.clusters, value=F)] <- "H1"
hm.clusters[grep("C3", hm.clusters, value=F)] <- "H2"
hm.clusters[grep("C6", hm.clusters, value=F)] <- "H3"
hm.clusters[grep("C8", hm.clusters, value=F)] <- "H4"
hm.clusters[grep("C1|C4|C5|C7", hm.clusters, value=F)] <- "N"
ML_Cluster <- ML_Cluster %>% 
  dplyr::mutate(Cluster=factor(hm.clusters, levels=c("H1","H2","H3","H4","N")))

# Train Random Forest classifier for HAND clusters.
ML_Cluster_Options <- trainControl(method="repeatedcv", number=3, repeats=10, classProbs=T)
ML_Cluster_RFModel <- train(Cluster~., dplyr::select(ML_Cluster, Cluster, dplyr::matches("AAIndex")),
                            method="rf", trControl=ML_Cluster_Options, tuneLength=2)
ML_Cluster_RFModel
```

```
## Random Forest 
## 
## 80 samples
##  3 predictor
##  5 classes: 'H1', 'H2', 'H3', 'H4', 'N' 
## 
## No pre-processing
## Resampling: Cross-Validated (3 fold, repeated 10 times) 
## Summary of sample sizes: 53, 54, 53, 54, 52, 54, ... 
## Resampling results across tuning parameters:
## 
##   mtry  Accuracy   Kappa    
##   2     0.9391229  0.9056450
##   3     0.9404491  0.9077469
## 
## Accuracy was used to select the optimal model using  the largest value.
## The final value used for the model was mtry = 3.
```

```
ggRF(caret_rf_model=ML_Cluster_RFModel$finalModel, num_nodes="median", lastColorGrey=T)
```

```
saveCurrentGraphicPDF("./figures/Figure3B.pdf", width=6, height=6)
```

## 4.2 Important amino acids for each of the HAND clusters.

```
# Calculate amino acid frequencies per patient
ML_Data_AA <- machineLearning.DataFormat(
  ml.matrix=machineLearning.Matrix(df.hand$"Sequence.AA", analysisType="boolian"),
  ml.metadata=df.hand,
  colNames.from=c("Reference_PMID","LANL_Patient.Id","Clinical.Status","Sample.Tissue.Category"), 
  colNames.to=c("StudyID","PatientID","NeuroOutcome","SampleTissue"), 
  outcomeLabelName="NeuroOutcome", 
  sourceLabelName="SampleTissue", 
  pp="none"
)
ML_Data_AA <- ML_Data_AA$MLDataFrame %>% dplyr::select(dplyr::matches("_mean"))
ML_Data_AA <- predict(caret::preProcess(ML_Data_AA, method=c("zv","nzv")), ML_Data_AA)

# Assign HAND clusters
ML_Data_AA <- data.frame("HANDCluster"=predict(ML_Cluster_RFModel, ML_Data$"MLDataFrame", type="raw"), ML_Data_AA)

# Important amino acids for each of the HAND clusters
ML_VarImp_AA <- caret::filterVarImp(ML_Data_AA[,-1], ML_Data_AA$"HANDCluster") %>%
  dplyr::mutate(AA=rownames(.)) %>%
  tidyr::gather(HANDCluster, Importance, -AA) %>%
  dplyr::filter(Importance > 0.9) 
ML_Data_AA <- dplyr::select(ML_Data_AA, HANDCluster, dplyr::one_of(sort(unique(ML_VarImp_AA$AA))))

# Heatmap
set.seed(1)
npg_colors <- c(gsub("FF","",pal_npg("nrc")(10)), "#808080")
hm.aa <- Heatmap(
  as.matrix(dplyr::select(ML_Data_AA, -HANDCluster)), 
  name="AA Frequency",
  col=circlize::colorRamp2(c(0, 0.5, 1), c("#56B4E9", "grey90", "#E69F00")),
  cluster_columns=F,
  show_column_dend=F,
  split=ML_Data_AA$"HANDCluster",
  gap=unit(2, "mm")
)
ha.aa <- rowAnnotation(
  df=dplyr::select(ML_Data_AA, HANDCluster),
  col=list(HANDCluster=c("H1"=npg_colors[1], "H2"=npg_colors[2], "H3"=npg_colors[3], 
                         "H4"=npg_colors[4],"N"=npg_colors[11]))
)
draw(ha.aa + hm.aa, padding=unit(c(2, 2, 2, 2), "mm"), row_sub_title_side="left")
```

```
saveCurrentGraphicPDF("./figures/SupplementaryFigure4.pdf", width=6, height=8)
```

## 4.3 Characterize the HAND clusters by referencing the HAND database.

```
df.handdb <- dataImportAndCombine(
  fileName.Alignment.FASTA=system.file("C2V3C3_HANDDatabase_AA.fasta", package="HANDPrediction"),
  fileName.HIVNeuroMetadata.CSV=system.file("HANDDatabase.csv", package="HANDPrediction"),
  fileName.SampleTissueCategoryDesignSheet.CSV=system.file(
    "HANDDatabase_SampleTissue_DesignSheet.csv", package="HANDPrediction"
    ),
  colName.SequenceID="Sequence..Accession.Number",
  colName.Sample.Tissue="Sampling..Tissue",
  colName.Clinical.Status="Patient..HAND.Status"
)
ML_Data_HANDDB <- machineLearning.DataFormat(
  ml.matrix=machineLearning.Matrix(df.handdb$"Sequence.AA"),
  ml.metadata=df.handdb,
  colNames.from=c("SEQUENCE.PMID","Patient..Code","Clinical.Status","Sample.Tissue.Category"), 
  colNames.to=c("StudyID","PatientID","HANDStatus","SampleTissue"), 
  outcomeLabelName=NULL, sourceLabelName="SampleTissue",
  pp="external", 
  pp.by.sequence=ML_Data$"MLDataPreProcessing_BySequence", 
  pp.by.patient=ML_Data$"MLDataPreProcessing_ByPatient"
)
ML_Pred_HANDDB <- dplyr::bind_cols(
  ML_Data_HANDDB$"MLMetadataDF",
  data.frame("PredictedOutcome"=predict(ML_Cluster_RFModel, ML_Data_HANDDB$"MLDataFrame", type="raw"),
             predict(ML_Cluster_RFModel, ML_Data_HANDDB$"MLDataFrame", type="prob"))
)
ML_Pred_HANDDB_Sankey <- ML_Pred_HANDDB %>% 
  dplyr::filter(HANDStatus %in% c("No HAND","HAD","HAD + HIVE","HIVE"))
sankey_colors <- npg_colors[c(8,3,4,11,5,1,2,6,11)]
gvSankey(
  reference=ML_Pred_HANDDB_Sankey$"HANDStatus", 
  prediction=ML_Pred_HANDDB_Sankey$"PredictedOutcome",
  colors_link=sankey_colors
)
```

# 5 Estimating the global burden of HAND

## 5.1 Los Alamos HIV Sequence Database.

```
df.lanl <- dataImportAndCombine(
  fileName.Alignment.FASTA=system.file("C2V3C3_LANL_AA.fasta", package="HANDPrediction"),
  fileName.HIVNeuroMetadata.CSV=system.file("LANL.zip", package="HANDPrediction"),
  fileName.SampleTissueCategoryDesignSheet.CSV=system.file(
    "LANL_SampleTissue_DesignSheet.csv", package="HANDPrediction"
    ),
  colName.SequenceID="Accession",
  colName.Sample.Tissue="Sample.Tissue"
)
ML_Data_LANL <- machineLearning.DataFormat(
  ml.matrix=machineLearning.Matrix(df.lanl$"Sequence.AA"),
  ml.metadata=df.lanl,
  colNames.from=c("Pubmed.ID","Patient.Id","Sample.Tissue.Category","Georegion","Country","Viral.load","CD4.count"), 
  colNames.to=c("StudyID","PatientID","SampleTissue","Georegion","Country","ViralLoad","CD4"), 
  outcomeLabelName=NULL, sourceLabelName="SampleTissue",
  pp="external", 
  pp.by.sequence=ML_Data$"MLDataPreProcessing_BySequence", 
  pp.by.patient=ML_Data$"MLDataPreProcessing_ByPatient"
)
ML_Pred_LANL <- dplyr::bind_cols(
  ML_Data_LANL$"MLMetadataDF",
  data.frame("PredictedOutcome"=predict(ML_Cluster_RFModel, ML_Data_LANL$"MLDataFrame", type="raw"),
             predict(ML_Cluster_RFModel, ML_Data_LANL$"MLDataFrame", type="prob"))
)
```

## 5.2 Global burden of HAND.

```
# Pie chart.
Fig.5A <- ggPie(ML_Pred_LANL$PredictedOutcome, colorSet=npg_colors[c(1:4,11)]) + theme(legend.position="top")

# Bar chart split by georegions.
ML_Pred_LANL_Georegion <- ML_Pred_LANL
ML_Pred_LANL_Georegion$"Georegion" <- as.character(ML_Pred_LANL_Georegion$"Georegion")
ML_Pred_LANL_Georegion$"Georegion"[which(ML_Pred_LANL_Georegion$"Georegion"=="Asia")] <- "Asia/Middle-East/Oceania"
ML_Pred_LANL_Georegion$"Georegion"[which(ML_Pred_LANL_Georegion$"Georegion"=="Middle-East")] <- "Asia/Middle-East/Oceania"
ML_Pred_LANL_Georegion$"Georegion"[which(ML_Pred_LANL_Georegion$"Georegion"=="Oceania")] <- "Asia/Middle-East/Oceania"
ML_Pred_LANL_Georegion$"Georegion"[which(ML_Pred_LANL_Georegion$"Georegion"=="AFR SSA")] <- "Sub-Saharan Africa"
ML_Pred_LANL_Georegion <- ML_Pred_LANL_Georegion %>% 
  dplyr::group_by(Georegion, PredictedOutcome) %>%
  dplyr::summarise(Count=n()) %>%
  dplyr::mutate(Freq=Count/sum(Count)) %>%
  dplyr::filter(Georegion != "South America")
Fig.5B <- ggplot(ML_Pred_LANL_Georegion, aes(x=Georegion, y=Freq, fill=PredictedOutcome)) +
  geom_bar(stat="identity", position="fill") + xlab(NULL) + ylab(NULL) +
  scale_fill_manual(values=npg_colors[c(1:4,11)], guide=guide_legend(title=NULL)) +
  scale_y_reverse(labels=scales::percent) +
  coord_flip() +
  theme_Publication() + theme(legend.position="top", legend.direction="horizontal")
Fig.5 <- cowplot::plot_grid(Fig.5A, Fig.5B, labels="AUTO", label_size=20, nrow=1, rel_widths=c(3,5), align="none")
savePDF(Fig.5, "./figures/Figure5.pdf", width=12, height=6)
```

## 5.3 Correlation of predicted HAND clusters and HIV-specific parameters.

```
options(scipen=0)
cowplot::plot_grid(
  ggplot(ML_Pred_LANL, aes(x = PredictedOutcome, y = ViralLoad, fill=PredictedOutcome)) +
    geom_violin(trim = F) +
    stat_summary(fun.y=median, geom = "point", color="white", shape = "-", size = 10) +
    ggsignif::geom_signif(
      comparisons = as.list(as.data.frame(combn(paste0("H", 1:4), 2), stringsAsFactors=F)),
      step_increase = 0.075, tip_length = 0, map_signif_level = F
    ) +
    xlab(NULL) + ylab("Viral load") + scale_y_log10() +
    scale_fill_manual(values=npg_colors[c(1:4,11)], guide=guide_legend(title=NULL)) +
    theme_Publication(),
  ggplot(ML_Pred_LANL, aes(x = PredictedOutcome, y = CD4,fill=PredictedOutcome)) +
    geom_violin(trim = F) +
    stat_summary(fun.y=median, geom = "point", color="white", shape = "-", size = 10) +
    ggsignif::geom_signif(
      comparisons = as.list(as.data.frame(combn(paste0("H", 1:4), 2), stringsAsFactors=F)),
      step_increase = 0.075, tip_length = 0, map_signif_level = F
    ) +
    xlab(NULL) + ylab("CD4 T-cell count") + scale_y_log10(breaks=c(10, 100, 1000)) +
    scale_fill_manual(values=npg_colors[c(1:4,11)], guide=guide_legend(title=NULL)) +
    theme_Publication(),
  labels = "AUTO", label_size = 20, nrow = 1, align = "none"
)
```

```
saveCurrentGraphicPDF("./figures/SupplementaryFigure5.pdf", width=12, height=6)
pairwise.wilcox.test(ML_Pred_LANL$ViralLoad, ML_Pred_LANL$PredictedOutcome, p.adjust.method = "BH", paired = F)
```

```
## 
##  Pairwise comparisons using Wilcoxon rank sum test 
## 
## data:  ML_Pred_LANL$ViralLoad and ML_Pred_LANL$PredictedOutcome 
## 
##    H1   H2   H3   H4  
## H2 0.42 -    -    -   
## H3 0.83 0.83 -    -   
## H4 0.83 0.83 0.90 -   
## N  0.42 0.83 0.83 0.83
## 
## P value adjustment method: BH
```

```
pairwise.wilcox.test(ML_Pred_LANL$CD4, ML_Pred_LANL$PredictedOutcome, p.adjust.method = "BH", paired = F)
```

```
## 
##  Pairwise comparisons using Wilcoxon rank sum test 
## 
## data:  ML_Pred_LANL$CD4 and ML_Pred_LANL$PredictedOutcome 
## 
##    H1     H2     H3     H4    
## H2 0.1472 -      -      -     
## H3 0.0072 0.2591 -      -     
## H4 0.8062 0.4379 0.1472 -     
## N  0.4379 0.3547 0.0141 0.8265
## 
## P value adjustment method: BH
```

# 6 Closing

## 6.1 Session info.

```
sessionInfo()
```

```
## R version 3.4.1 (2017-06-30)
## Platform: x86_64-w64-mingw32/x64 (64-bit)
## Running under: Windows 7 x64 (build 7601) Service Pack 1
## 
## Matrix products: default
## 
## locale:
## [1] LC_COLLATE=Japanese_Japan.932  LC_CTYPE=Japanese_Japan.932   
## [3] LC_MONETARY=Japanese_Japan.932 LC_NUMERIC=C                  
## [5] LC_TIME=Japanese_Japan.932    
## 
## attached base packages:
##  [1] grid      splines   parallel  stats     graphics  grDevices utils    
##  [8] datasets  methods   base     
## 
## other attached packages:
##  [1] ComplexHeatmap_1.14.0     xgboost_0.6-4            
##  [3] plyr_1.8.4                gbm_2.1.3                
##  [5] survival_2.41-3           randomForest_4.6-12      
##  [7] kernlab_0.9-25            bindrcpp_0.2             
##  [9] caret_6.0-76              lattice_0.20-35          
## [11] googleVis_0.6.2           ggsci_2.7                
## [13] RColorBrewer_1.1-2        dplyr_0.7.2.9000         
## [15] purrr_0.2.3               readr_1.1.1              
## [17] tidyr_0.6.3               tibble_1.3.3             
## [19] ggplot2_2.2.1             tidyverse_1.1.1          
## [21] HANDPrediction_0.0.0.9000 devtools_1.13.3          
## [23] doParallel_1.0.10         iterators_1.0.8          
## [25] foreach_1.4.3            
## 
## loaded via a namespace (and not attached):
##   [1] circlize_0.4.1       readxl_1.0.0         uuid_0.1-2          
##   [4] backports_1.1.0      igraph_1.1.2         lazyeval_0.2.0      
##   [7] digest_0.6.12        htmltools_0.3.6      viridis_0.4.0       
##  [10] magrittr_1.5         memoise_1.1.0        cluster_2.0.6       
##  [13] modelr_0.1.1         R.utils_2.5.0        officer_0.1.5       
##  [16] colorspace_1.3-2     rvest_0.3.2          ggrepel_0.6.12      
##  [19] haven_1.1.0          jsonlite_1.5         optimbase_1.0-9     
##  [22] roxygen2_6.0.1       lme4_1.1-13          bindr_0.1           
##  [25] zoo_1.8-0            ape_4.1              glue_1.1.1          
##  [28] rvg_0.1.4            gtable_0.2.0         MatrixModels_0.4-1  
##  [31] seqinr_3.4-5         GetoptLong_0.1.6     car_2.1-5           
##  [34] shape_1.4.2          DEoptimR_1.0-8       prabclus_2.2-6      
##  [37] SparseM_1.77         scales_0.4.1         mvtnorm_1.0-6       
##  [40] ggthemes_3.4.0       Rcpp_0.12.12         viridisLite_0.2.0   
##  [43] xtable_1.8-2         units_0.4-5          mclust_5.3          
##  [46] foreign_0.8-69       stats4_3.4.1         survey_3.32-1       
##  [49] httr_1.2.1           fpc_2.1-10           modeltools_0.2-21   
##  [52] flexmix_2.3-14       pkgconfig_2.0.1      rJava_0.9-8         
##  [55] R.methodsS3_1.7.1    manipulate_1.0.1     nnet_7.3-12         
##  [58] labeling_0.3         rlang_0.1.1.9000     reshape2_1.4.2      
##  [61] munsell_0.4.3        cellranger_1.1.0     tools_3.4.1         
##  [64] rpanel_1.1-3         ade4_1.7-8           broom_0.4.2         
##  [67] evaluate_0.10.1      stringr_1.2.0        yaml_2.1.14         
##  [70] ggtree_1.8.1         ModelMetrics_1.1.0   knitr_1.17          
##  [73] robustbase_0.92-7    zip_1.0.0            dendextend_1.5.2    
##  [76] ggraph_1.0.0         nlme_3.1-131         whisker_0.3-2       
##  [79] mime_0.5             quantreg_5.33        R.oo_1.21.0         
##  [82] xml2_1.1.1           compiler_3.4.1       pbkrtest_0.4-7      
##  [85] ReporteRs_0.8.8      png_0.1-7            ggsignif_0.4.0      
##  [88] e1071_1.6-8          treeio_1.0.2         tweenr_0.1.5        
##  [91] DescTools_0.99.21    stringi_1.1.5        forcats_0.2.0       
##  [94] gdtools_0.1.5        trimcluster_0.1-2    Matrix_1.2-10       
##  [97] commonmark_1.2       psych_1.7.5          nloptr_1.0.4        
## [100] stringdist_0.9.4.6   GlobalOptions_0.0.12 cowplot_0.8.0       
## [103] data.table_1.10.4    httpuv_1.3.5         R6_2.2.2            
## [106] Rmisc_1.5            gridExtra_2.2.1      codetools_0.2-15    
## [109] boot_1.3-19          MASS_7.3-47          assertthat_0.2.0    
## [112] rjson_0.2.15         rprojroot_1.2        withr_2.0.0         
## [115] mnormt_1.5-5         diptest_0.75-7       mgcv_1.8-18         
## [118] expm_0.999-2         hms_0.3              udunits2_0.13       
## [121] tableone_0.8.1       class_7.3-14         minqa_1.2.4         
## [124] ReporteRsjars_0.0.2  rmarkdown_1.6        rvcheck_0.0.9       
## [127] ggforce_0.1.1        shiny_1.0.3          lubridate_1.6.0     
## [130] base64enc_0.1-3
```

## 6.2 Save the workspace.

```
save.image(file="HANDPrediction_Workspace.RData")
```
